# Membrane repair following filtroporation-induced cell permeabilization

**DOI:** 10.1016/j.isci.2025.114317

**Published:** 2025-12-05

**Authors:** Isaura M. Frost, Ruby Sims, Anya Yakimenko, Rachel Ma, Maximilian Floridia, Ruth A. Foley, Emily C. Duggan, Kelly Y. Cai, Kelsey Jorgensen, Emily Skuratovsky, Paul S. Weiss, Steven J. Jonas

**Affiliations:** 1 Department of Bioengineering, University of California, Los Angeles, Los Angeles, CA 90095, USA; 2 UCLA Medical Scientist Training Program, David Geffen School of Medicine, University of California, Los Angeles, Los Angeles, CA 90095, USA; 3 Department of Pediatrics, David Geffen School of Medicine, University of California, Los Angeles, Los Angeles, CA 90095, USA; 4 Department of Chemistry and Biochemistry, University of California, Los Angeles, Los Angeles, CA 90095, USA; 5 Department of Molecular, Cell and Developmental Biology, University of California, Los Angeles, Los Angeles, CA 90095, USA; 6 Department of Anthropology, University of Kansas, Lawrence, KS 66045, USA; 7 Eli & Edythe Broad Center of Regenerative Medicine and Stem Cell Research, University of California, Los Angeles, Los Angeles, CA 90095, USA; 8 California NanoSystems Institute, University of California, Los Angeles, Los Angeles, CA 90095, USA; 9 Department of Materials Science and Engineering, University of California, Los Angeles, Los Angeles, CA 90095, USA; 10 Children’s Discovery and Innovation Institute, University of California, Los Angeles, Los Angeles, CA 90095, USA

**Keywords:** cell biology, omics

## Abstract

Mechanical disruption of cell membranes to enable intracellular delivery has been gaining traction as a methodology. Here, we show that a mechanical disruption-based strategy, filtroporation (FP), can be applied to edit the beta globin gene efficiently in human hematopoietic stem and progenitor cells. Gene expression analyses from RNA-Seq datasets demonstrate that electroporation (EP) yields greater transcriptional changes compared to FP globally, and gene pathway enrichment analyses suggest EP promotes stem cell differentiation while FP promotes cell division. Membrane repair occurs within 30 s of disruption by FP, and calcium signaling plays a key role in membrane repair in this context. Studies with fluorescently tagged membrane repair proteins, GRAF1, SNAP23, and CHMP4B, implicate the involvement of three mechanisms to reconstitute the cell barrier following FP. This work supports the evidence that the choice of intracellular delivery method affects transcriptional stem cell profile, and that membrane repair after mechanical disruption is a fast, multi-pathway process.

## Highlights

• Filtroporation enables delivery of CRISPR/Cas9 reagents and editing at the beta globin locus
• Filtroporation causes fewer transcriptional changes than electroporation
• Cells repair their membranes within 30 s of filtroporation
• Multiple membrane repair mechanisms are involved following filtroporation

## Graphical abstract

**Figure:**
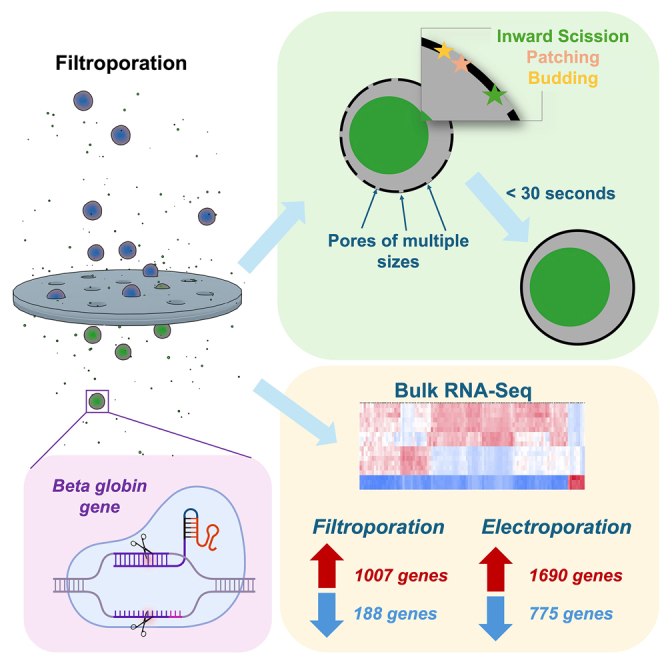

## Introduction

Achieving effective intracellular delivery of biomolecular cargoes by the transient permeabilization of cellular membrane barriers represents an ongoing challenge in both research and clinical settings.^1^^,^^2^^,^^3^ Several delivery methodologies are under development that may enable more efficient manufacturing of next-generation cell-based therapies and do not require the use of viral vectors.^4^^,^^5^^,^^6^ We and other groups have demonstrated that the mechanical deformation of cells results in the formation of transient pores along their plasma membranes, enabling the intracellular diffusion of biomolecular cargoes.^7^^,^^8^^,^^9^^,^^10^^,^^11^^,^^12^^,^^13^ Notable examples of cell permeabilization methods via mechanical membrane disruption from our group include acoustofluidic sonoporation^7^ and lipid-bicelle-coated microfluidic devices.^8^ Similar cell squeezing approaches have also been shown to be applicable in other settings, such as in stem cell neuronal differentiation protocols.^14^ These mechanical deformation techniques usually require cells to be compressed to *ca*. 30–80% of their average free diameter to be rendered porous. Biophysically manipulated cell populations display dramatically fewer side effects, such as minimal transcriptional perturbations, decreased cytokine secretion, and maintenance of functionality *in vivo* in T cells, as compared to those treated via bulk electroporation (EP).^15^^,^^16^ Alternatives to bulk EP have been devised that minimize its detrimental effects on cell function, such as by the incorporation of a nanostructured electro-injection platform.^17^ However, this platform may only process 200,000 cells per experiment and thus may be difficult to scale. Although permeabilization induced by cell deformation has shown great promise, the mechanisms underlying the formation and resealing of membrane discontinuities or pores as a consequence of cell deformation have not been systematically tested and are not well understood.^6^

These transfection platforms are promising but share one drawback, which is their requirement for specialized equipment. To address this issue, we recently reported a straightforward filtroporation (FP) strategy that enables the rapid deformation of cells in a scalable, highly efficient, and minimally toxic manner.^18^ As cells are passed through the pores of track-etched poly(ethylene terephthalate) (PET) cell culture inserts that are modified with anti-fouling surface chemistries, they become temporarily permeabilized. Intracellular delivery of expression plasmids, dextrans, and clustered regularly interspaced short palindromic repeats (CRISPRs)/Cas9 ribonucleoproteins (RNPs) has been demonstrated in immortalized Jurkat cells and human CD34^+^ hematopoietic stem and progenitor cells (HSPCs).^18^ Membrane repair kinetics following permeabilization by mechanical squeezing have begun to be explored in microfluidic embodiments of this method,^19^ and showed that membranes recover within a few seconds of permeabilization by squeezing. Mechanoporation systems recapitulate the natural phenomenon of cell migration through narrow capillaries^20^ while taking advantage of the resulting temporary membrane permeability to deliver biomolecular payloads. Developing an improved understanding of how cells respond to these treatments will offer key insight into how to best apply mechanical transfection methods at the bench and at clinical scales.

Evidence in the literature suggests that membrane repair following mechanical disruption is calcium dependent^21^^,^^22^^,^^23^ and may take place through a variety of mechanisms.^24^ Previous work on cell-squeezing platforms corroborates this idea, showing that the addition of CaCl_2_ to transfection media led to decreased transfection efficiency for primary murine T cells,^19^ likely due to improved membrane repair in the setting of higher calcium concentration. All known membrane repair mechanisms involve calcium signaling, including exocytosis or patching, inward scission, and outward budding and fission of the cell membrane.^24^ Patching has been proposed to be active in repairing large membrane defects (>200 nm) and occurs when several small intracellular vesicles first merge with one another and subsequently dock onto the plasma membrane. This process, such as the exocytotic secretion of intracellular components, is mediated by soluble *N*-ethylmaleimide-sensitive factor (NSF) attachment protein receptors (SNAREs) that comprise a large family of proteins involved in vesicle fusion.^24^^,^^25^ Critical to its function is the vesicle associate membrane protein 7 (VAMP7), which has been shown to promote both patching and lysosomal exocytosis mechanisms, and synaptosome associated protein 23 (SNAP23), a key component of SNAREs.^24^ The budding mechanism, thought to be active in repairing smaller defects (<100 nm), is instead mediated by the endosomal sorting complexes required for transport (ESCRT) proteins, particularly ESCRT-III. Depletion of its core components, such as charged multivesicular protein 4B (CHMP4B),^26^ or of vacuolar protein sorting-associated protein 4B (VPS4B), which controls assembly and disassembly of the ESCRT-III complex, leads to impairment in membrane repair.^24^ One additional mechanism involves the inward budding of the damaged membrane, followed by fusion of this vesicle with an endosome or lysosome, a process also known as membrane scission toward the cytosol. A key protein involved in this process is GTPase regulator associated with focal adhesion kinase 1 (GRAF1), also known as Rho GTPase-activating protein 26 (ARHGAP26), which promotes clathrin-dependent endocytosis. This mechanism is also thought to be involved in the repair of membrane defects in the 50–100 nm range.^24^ To our knowledge, studies that elucidate which of these mechanisms are involved in membrane repair after physical or electrical manipulation remain limited, but would be valuable to shed light on the size of membrane pores and the potential impact of these treatments on target cells, to devise future platforms, or to study membrane repair in this context.

In this work, we further build upon our filtroporation platform and demonstrate its capability in delivering clinically relevant Cas9 ribonucleoprotein (RNP) complexes for editing at the human beta globin (HBB) locus. Transcriptomic analysis of cell populations following FP reveals differences in global differential expression as well as in key pathways involved in hematopoiesis and the cell cycle compared to nucleofection, aligning with findings from Sharei and colleagues.^16^ We report that membrane porosity decreases dramatically within 30 s of permeabilization by FP, and show that calcium signaling is a critical component for cell survival after FP-mediated mechanical permeabilization. Additionally, our membrane repair studies suggest that pores created by FP are of sizes ranging from tens of nanometers to microns, supporting the idea that cargoes of a variety of sizes may be delivered by this method. Our work also suggests filtroporation could be an economical and easily implementable method for researchers interested in studying membrane repair after mechanical cell membrane damage.

## Results

### Filtroporation causes fewer transcriptional changes in human hematopoietic stem and progenitor cells compared to electroporation

**Figure 1:**
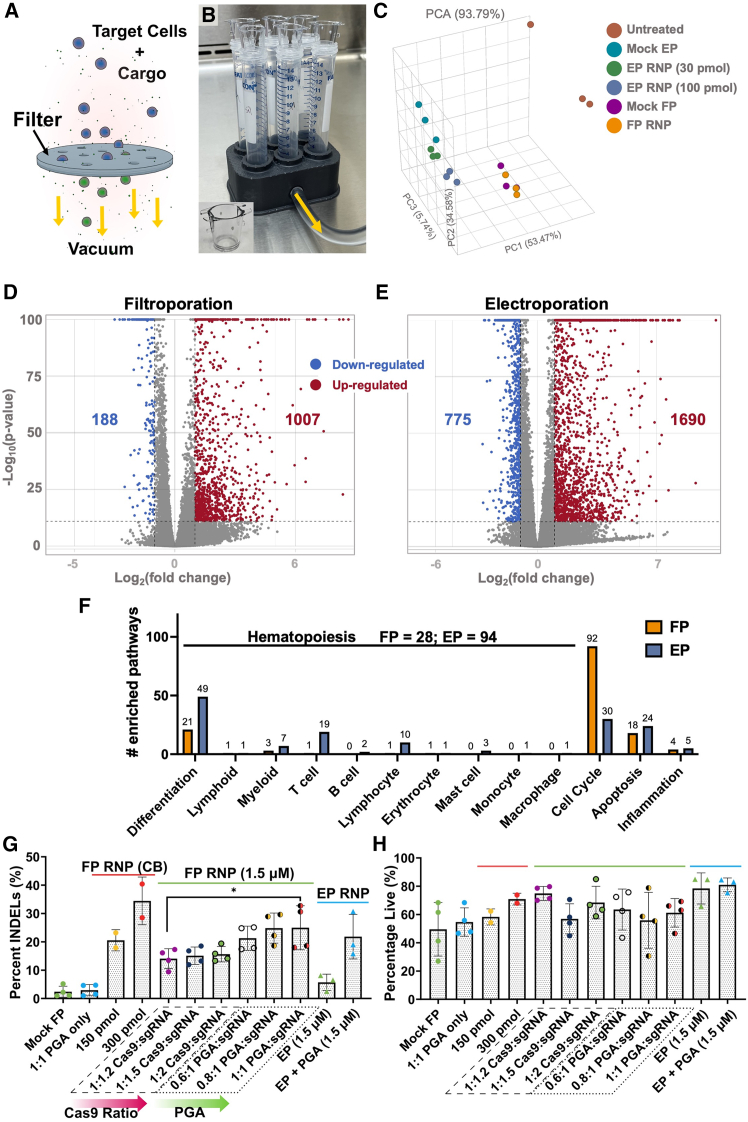
Delivery and editing at the beta globin locus and RNA-Seq following the filtroporation-mediated permeabilization of human hematopoietic stem and progenitor cells (HSPCs) (A) Schematic of filtroporation-induced cell deformation and permeabilization. (B) Digital image of filtroporation system with 6 parallel reactions. Inset displays digital image of cell culture insert used for filtroporation. (C) Principal component analysis from RNA sequencing (RNA-Seq) studies. (D and E) Volcano plots of differentially expressed genes based on the RNA-Seq analysis of HSPCs 3 h post filtroporation (FP) or electroporation (EP). CD55-targeting Cas9 ribonucleoproteins (RNPs) were used as a model cargo. (F) Quantified pathway analysis matching keywords for each biological process category. (G) Percentage of insertions and deletions (INDELs) as determined by PCR, Sanger sequencing, and tracking of INDELs by the decomposition (TIDE) analysis of genomic DNA extracted from HSPCs pre-stimulated for 48 h at 4–5 days post-FP with RNPs designed to target the human beta globin (HBB) gene. Sources of HSPCs were either cord blood (CB) or peripheral blood mobilized HSPCs (PBSCs). Samples were either treated in the absence of cargo (Mock FP), FP-treated only with polyglutamic acid (PGA), FP-treated with RNPs formulated with increasing ratios of Cas9:sgRNA (1:1.2 to 1:2), or FP-treated with RNPs plus varying amounts of PGA (varied from 0.6:1 to 1:1 PGA:sgRNA volume ratios). Unless otherwise noted, the Cas9:sgRNA ratio was 1:1.2. (H) Viabilities of HSPCs corresponding to treatments in (G) at 24 h post-FP as determined by acridine orange/propidium iodide fluorescent staining. Data are shown for *n* ≥ 2 biologically independent donors from at least two independent experiments. (∗*p* < 0.05) RNA-Seq data are obtained from triplicate samples in experiments with PBSCs from a single donor. (∗*p* < 0.05). See also Figures S1 and S2. Data are shown as mean ± standard deviation.

In our previous work, we demonstrated CRISPR-mediated knockout of a model gene by delivery of RNPs by filtroporation (Figures 1A and 1B) in HSPCs.^18^ To understand the impact of filtroporation on stem cell biology, we performed the next-generation sequencing of RNA extracted from peripheral blood-mobilized stem cells (PBSCs) 3 h post-filtroporation, delivering Cas9 RNP targeting the CD55 gene. We chose this time point to elucidate changes that occurred shortly after treatment, given our interest in membrane repair while still allowing sufficient time for transcription to occur. Although these data may show transient transcriptomic changes, we believe it is valuable to understand the effects of these treatments within short time frames as initial changes may be of interest in the setting of differentiation or cell survival, for instance; additionally, later time points have already been reported elsewhere.^16^^,^^17^ Nucleofection of the same cell population was performed concurrently as a control using two different concentrations of RNP: 30 pmol, corresponding to the same final concentration as used in FP (1.5 μM), and 100 pmol, corresponding to the amount used in a typical EP experiment. First, we analyzed differential expression in RNA-Seq datasets comparing treated to untreated controls (FP vs. untreated and EP vs. untreated; here, FP and EP include RNP cargo at typical experimental concentrations, and the same untreated control group and cell batch were used across conditions). Principal component plots (Figure 1C) show similarly treated samples cluster together, even when cargo is added (Mock EP/FP vs. EP/FP RNP). We generated volcano plots displaying statistically significantly differentially expressed genes and found that FP treatment caused the upregulation of 1007 genes and downregulation of 188 (Figure 1D), while EP upregulated 1690 genes and downregulated 775 (Figure 1E). A heatmap of the significantly differentially expressed genes common to both comparisons (FP vs. untreated and EP vs. untreated) shows that cells from FP-treated samples are more similar to untreated controls (Figure S1C), similar to reports by Sharei and co-workers on a microfluidic cell squeezing platform.^16^ These data suggest that EP may skew cells from their native transcriptomic profile to a greater extent than FP. Pathway enrichment analysis revealed that the top 20 enriched pathways after FP treatment were all related to the cell cycle and mitosis (Figures 1F and S1A), whereas, after EP, pathways related to hemopoiesis and lymphocytosis were highly enriched (Figures 1F and S1B). We found that performing pathway analyses was more relevant than analyzing transcript counts for individual genes given that even genes well-known to be inflammatory markers can also have diverse roles within the cell milieu, especially within a potentially heterogeneous sample in the context of bulk RNA sequencing (e.g., IL1b was significantly enriched in 355 pathways, however, only 2 of those pathways were related to inflammation). We searched through significantly enriched pathways (FDR <0.01) using hematopoietic lineage-related keywords and found 94 significantly enriched pathways within EP-treated samples, while 28 were enriched in FP-treated samples (see Tables S1 and S2). From the EP samples, 49 pathways were related to differentiation, and 31 matched “T cell,” “B cell,” or “lymphocyte,” suggesting EP may promote HSPC differentiation into lymphoid cells, while only 21 and 2 pathways, respectively, were found matching these terms within FP samples. Filtroporation samples had 92 enriched pathways related to the cell cycle, while EP only had 30. Taken together, these data suggest that FP may strongly promote cell division, while EP strongly promotes differentiation, especially toward lymphoid lineage, within the first hours after treatment. Furthermore, EP enriched more apoptotic pathways than FP, with 24 and 18 enriched pathways, respectively. Inflammatory pathways were similar between the two treatment groups, with 4 enriched pathways for FP and 5 for EP (supplemental table from ShinyGO analysis is available for searches).

### Filtroporation enables gene editing at the beta globin locus

While our prior work demonstrated CD55 knockout,^18^ here, we focus on applying and optimizing filtroporation for modification at a clinically relevant genomic locus, human beta globin (HBB), using single guide RNA (sgRNA) constructs designed for application in HSPC-modified gene therapies by Kohn and co-workers.^15^^,^^27^ We optimized the conditions to maximize non-homologous end-joining (NHEJ)-mediated cutting in this system, measured as percent insertions or deletions (INDELs) by polymerase chain reaction (PCR), Sanger sequencing, and tracking of INDELs by decomposition (TIDE) analysis. Here, we pre-stimulated the cells for 48 h as reported for this sgRNA,^15^ and our testing confirmed that the pre-stimulation of HSPCs for 48 h was optimal (Figures S2A and S2C for 24 h pre-stimulation; Figures 1G and 1H for 48 h pre-stimulation). Our initial strategies to optimize KO efficiency at the 24 h pre-stimulation timepoint included the addition of an oligonucleotide commonly used as an EP enhancer (“electroporation enhancer” from Integrated DNA Technologies), a copolymer (Pluronic F-68) previously used as a delivery enhancer,^7^^,^^9^ and increasing RNP cargo concentrations. All these treatments yielded minimal improvements in INDEL frequencies (Figure S2A) and incurred significant toxicities (Figure S2C). Hematopoietic stem and progenitor cells from two different sources (cord blood, CB, or peripheral blood mobilized, PBSCs) were tested, with CB-derived cells displaying higher levels of INDELs (35 ± 8% for 300 pmol RNP amounts with Cas9 to sgRNA molar ratio of 1:1.2). However, limited cell numbers obtained from CB sources precluded further testing; thus, we turned to PBSCs for further optimization studies. Others have reported that increasing the molar excess of sgRNA when assembling RNPs leads to increased KO efficiencies due to the improved solubilization of the RNP complex, which tends to come out of solution when Cas9 and sgRNA are initially mixed.^28^

Alternatively, Marson and colleagues also tested the addition of various polymers to improve RNP solubility and found that premixing sgRNA with polyglutamic acid (PGA) resulted in similar increases in INDEL frequencies in samples transfected by EP.^28^ Inspired by this work, we tested the impact of changing the Cas9:sgRNA ratio as well as the addition of PGA at a fixed Cas9:sgRNA ratio of 1:1.2 (previously used in our experiments).^18^ We found that increasing the Cas9:sgRNA ratio from 1:1.2 to 1:2 led to slight increases in INDEL frequencies (not statistically significant), but the addition of PGA at amounts previously reported^28^ led to significant increases in percent INDELs (from 15 ± 4% to 25 ± 8%) at its highest amount of 1:1 PGA:sgRNA v/v in PBSCs (Figure 1G). Viabilities remained >50% as determined by acridine orange/propidium iodide staining at 24 h post-FP (Figure 1H), suggesting that the addition of excess sgRNA and PGA does not significantly alter cell viability. Interestingly, we observed that the cell viabilities for the PGA and RNP conditions are higher than for “Mock FP” conditions; these results are likely due to several factors, including variability in cell sources (e.g., different donors across experiments and conditions), as well as the fact that each filter is unique with random pore distributions due to their manufacturing process, potentially resulting in configurations that may have been harsher on cells in a particular experiment. Control EP experiments were performed in parallel with PBSC batches using a concentration of 1.5 μM HBB-targeting RNP (30 pmol), thus matching the RNP concentration used in FP experiments. We also tested the addition of PGA at the optimized reported concentration^28^ (0.8:1 PGA:sgRNA v/v) in the EP system. We found that FP outperforms EP in generating INDELs at the matched concentration (15 ± 4% with FP versus 7 ± 3% with EP), supporting our previous findings using CRISPR/Cas9 reagents designed for CD55 knockout.^18^ Interestingly, the addition of PGA appears to boost KO efficiency in EP to a greater extent than it does for FP (EP: 7 ± 3% without PGA to 19 ± 8% with PGA, a 2.7-fold increase; FP: 1.7-fold increase), suggesting that the mechanism by which PGA improves efficiencies may be more complex than simply solubilization (Figure S2B). Although the exact mechanism remains unclear, it is known that macromolecular delivery in EP is charge dependent, as negatively charged cargoes are pulled toward the positive electrode as the membrane is permeabilized by an electrical pulse.^5^^,^^10^ Thus, we hypothesize that the addition of a negatively charged polymer such as PGA, improves delivery outcomes by entangling with cargoes and “dragging” them along in solution. Finally, transfection yield is an important concept that reflects the fraction of the starting material that survives the treatment with the desired modification. The conditions with the highest yields were the 300 pmol CB samples, with a yield of 24.5%. Out of the PBSC population, the highest yields were obtained with the 1:1 PGA:sgRNA conditions at 13.7%.

### Cell membranes repair in seconds following filtroporation

**Figure 2:**
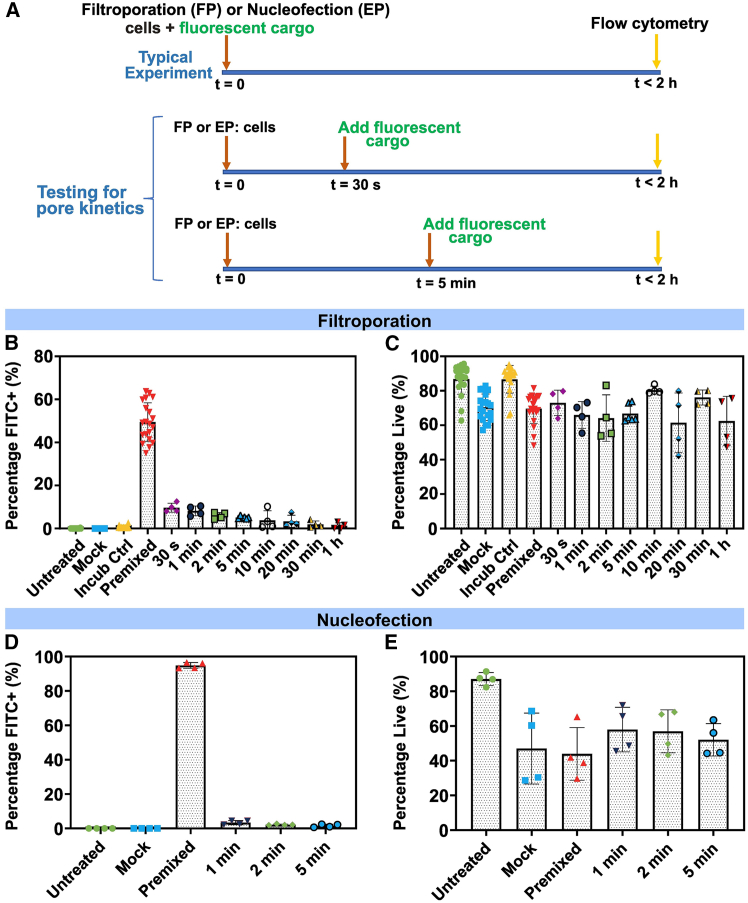
Timed delivery of fluorescently tagged dextran to Jurkat cells (A) Schematic of typical and timed delivery experiments performed. In a typical experiment, target cells are premixed with fluorescent cargo (fluorescein isothiocyanate dextran, FITC-Dex). In timed delivery experiments, cells are treated in delivery buffer without cargo while FITC-Dex is added at a later time point. Flow cytometry is performed within 2 h of treatment to determine delivery efficiency (percentage of cells positive for FITC). (B) Filtroporation of Jurkat cells with FITC-Dex; cargo was either premixed or added at the specified time point. Controls were untreated, mock filtroporated (FP without cargo) or incubated with FITC-Dex without FP (Incub Ctrl). (C) Viabilities determined by 4′,6-diamidino-2-phenylindole (DAPI) staining at the time of flow cytometry after FP experiments. (D) Nucleofection (EP) of Jurkat cells with FITC-Dex; cargo was either premixed or added at the specified time point. Controls were untreated or mock nucleofected (EP without cargo). (E) Viabilities determined by 4′,6-diamidino-2-phenylindole (DAPI) staining at the time of flow cytometry after EP experiments. Data are shown for *n* ≥ 2 independent experiments. See also Figures S3 and S4. Data are shown as mean ± standard deviation.

We designed a series of experiments to test how long the cell membrane remains permeable following treatment by FP. During FP, target cells are typically premixed with the desired cargo. To evaluate the timing of membrane repair, cargo is introduced to FP cells at specified time points ranging from 30 s to 1 h (Figure 2A). Here, we utilized a fluorescein isothiocyanate (FITC)-conjugated dextran molecule (average molecular weight 40 kDa) (FITC-Dex) as a model cargo for the rapid determination of delivery efficiency by flow cytometry. We hypothesized that if the cell membrane permeability generated by FP persists for longer than the time passed between FP and cargo introduction, we would observe delivery comparable to premixed controls, measured as the fraction of FITC^+^ cells at the time of flow cytometry. We observed a steep decline in delivery efficiency from 49 ± 9% for premixed controls, to less than 10 ± 2% when FITC-Dex cargo is introduced at 30 s (Figure 2B). This result suggests that membrane permeability sharply decreases within 30 s following FP. A consistent decrease in delivery efficiency follows for the remaining timepoints (1 min through 1 h), while untreated, mock FP (filtroporated without cargo), and incubation (incubated in FITC-Dex but not subjected to FP) controls show negligible background fluorescence. Viabilities determined by 4′,6-diamidino-2-phenylindole (DAPI) counterstaining at the time of flow cytometry remain greater than 60% for all conditions (Figure 2C). These findings indicate that cells begin to repair their membrane within 30 s after FP treatment, and thus cargo provided after this time point can no longer enter cells due to their decreased permeability. We compare these results to FITC-Dex delivered by the commercially available transfection method nucleofection (a type of electroporation and thus abbreviated as EP) and also observed a steep decrease in efficiency when cargo delivery is delayed by 1 min (from 95 ± 2% down to 3%). The sharp decline is similar to the one documented after FP and indicates that the permeability of cells sharply decreases within 1 min following EP (Figure 2D). Note that due to logistical limitations in performing nucleofection, which is conducted outside of the biosafety cabinet, it was not possible to deliver cargo less than 1 min after the cells were treated by EP. Viabilities for EP were lower than those of FP and remained between 25% and 70%, as determined by DAPI during flow cytometry (Figure 2E). These data suggest that membrane repair following both mechanical and electrical permeabilization is a process occurring within seconds of treatment, corroborating this same finding in the membrane repair literature.^24^

To test whether fluorescent cargo was entering the cells rather than attaching to the outer plasma membrane in these experiments, controls and treated cells were visualized with fluorescent microscopy (Figure S3). We observed uniformly fluorescent cells predominantly in FP or EP premixed conditions (Figures S3J–S3L), while conditions with delayed cargo delivery displayed patchy fluorescence with overlapping DAPI staining, indicating dead cells (Figures S3N–S3P). Although we exclude dead cells from our FITC^+^ gating during flow cytometry data analyses, some cells may not have been completely stained by DAPI at the time of flow cytometry and, therefore, may have been counted as live cells. However, when these same cells were taken to fluorescent imaging after flow cytometry, we observed that fluorescent cells in the delayed delivery conditions were co-stained by DAPI and were therefore not viable. This observation led us to draw tighter flow cytometry gates when analyzing the data displayed in Figure 2, as we concluded that the fluorescence observed in the delayed cargo experiments is likely predominantly present in dying cells with increased membrane permeability (Figure S4). It is also possible that because FP is a physical method, cell membranes may still be interacting with cargo several minutes after FP, but this cargo has not entered the cytoplasm and thus never becomes effectively delivered. Taken together, these data suggest that membrane repair occurs within 30 s or 60 s of permeabilization by FP and EP, respectively, and possibly within an even shorter time frame. Concerning practical applications, the results support the strategy of premixing cargo with cell populations to ensure delivery of biomolecular payloads and suggest that apparent delivery (positive fluorescent signal at flow cytometry) seen in delayed cargo experiments may be the result of biomolecules entering cells due to higher permeability secondary to cell death.

### Multiple membrane repair pathways are active following filtroporation-induced permeabilization

Next, we sought to understand the mechanisms involved in membrane repair after filtroporation treatment. In addition to gaining biological understanding, discovering these mechanisms could help elucidate the sizes of the pores formed on the cell surface upon permeabilization by FP. To study pore sizes, we initially performed FP and electroporation as a control, with polystyrene (PS) beads of narrow polydispersity and diameter in the range of pore sizes that we anticipated may be present. We assumed that, given that beads are solid, effectively incompressible spherical objects, they would only enter cells if the pores present on the plasma membrane were at least the same diameter as the beads. Our initial trial utilized PS beads 50 nm in diameter (Figures S5A–S5H); this size was chosen due to our previous success in delivering Cas9 ribonucleoproteins,^18^ which have previously been estimated to be 10–100 nm in hydrodynamic diameter in various buffers.^28^^,^^29^ To test whether the beads were attached to the outsides of cells, an incubation control (“Incubation Ctrl”) was also performed. We observed that the beads appeared to attach strongly to the surfaces of the cells and could not be removed even after several washing steps (Figure S5C). Furthermore, we found that the PS beads appeared to result in cell shearing during filtroporation, as evidenced by massive cell loss and low cell recovery (nearly no cell pellet seen) after experiments (Figures S5E and S5F). In EP controls, although fluorescence was observed (Figures S5G and S5H), it was not possible to differentiate from incubation controls and thus no conclusions about delivery could be drawn. Given these data, no further experiments were performed with PS beads.

**Figure 3:**
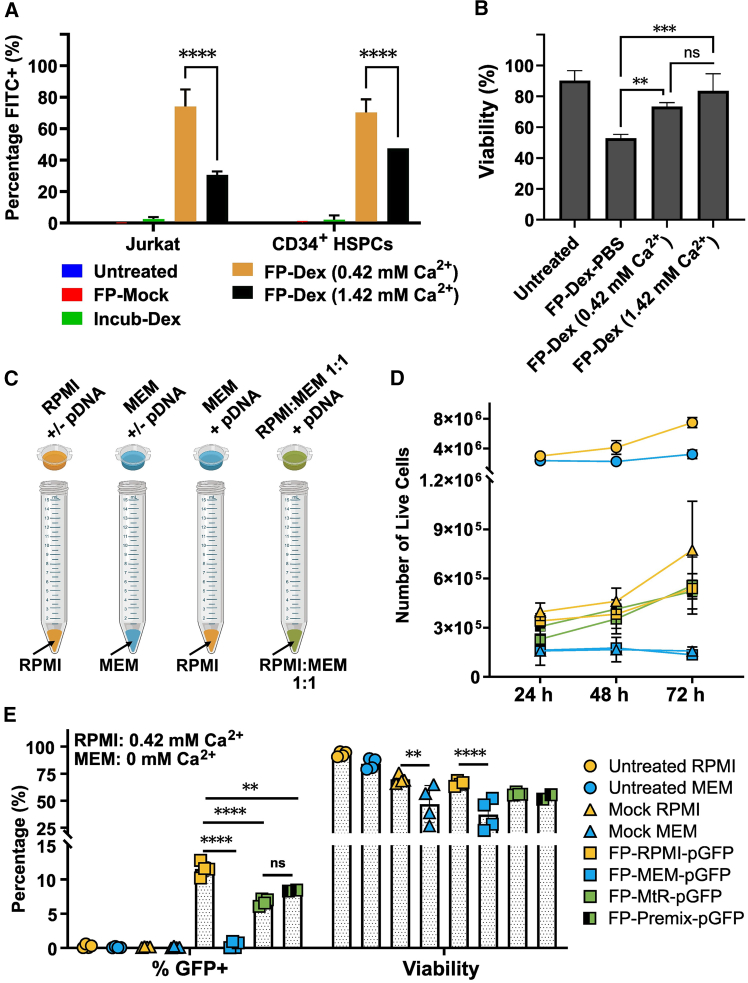
Calcium concentration is critical during transfection by filtroporation (A) Delivery efficiency determined by flow cytometry within 2 h of filtroporation of Jurkat or CD34^+^ bone marrow derived human hematopoietic stem and progenitor cells (HSPCs) with fluorescein isothiocyanate (FITC)-tagged dextran (FITC-Dex) in cell media with low calcium (Roswell Park Memorial Institute, RPMI), FP-Dex (0.42 mM Ca^2+^), or with additional 1 mM CaCl_2_ added, FP-Dex (1.42 mM Ca^2+^). Controls were either untreated, filtroporated without cargo (FP-Mock), or incubated in FITC-Dex without FP (Incub-Dex). (B) Viabilities of filtroporated Jurkat cells determined by 4′,6-diamidino-2-phenylindole (DAPI) at the time of flow cytometry. Cells were also transfected in phosphate buffered saline (PBS) as delivery buffer (FP-Dex-PBS). (C) Jurkat cells were filtroporated with green fluorescent protein (GFP)-encoding plasmid (pGFP) in different delivery buffers: RPMI, minimum essential media (MEM) containing no calcium, or a 1:1 mixture of RPMI and MEM (0.21 mM Ca^2+^). Media was introduced at the bottom of the collection tube such that cells subjected to filtroporation fell into either RPMI, MEM or RPMI:MEM 1:1 media. Results are displayed for cells at 24 h post-filtroporation (timepoint with highest expression). (D) Cell counts at 24–72 h post-filtroporation performed with acridine orange/propidium iodide fluorescent staining. (E) Percentage of GFP-positive cells (left) and cell viability (right) determined by DAPI staining at the time of flow cytometry. Mock RPMI/MEM: cells filtroporated without cargo in RPMI or MEM; FP-RPMI-pGFP: cells subjected to FP in RPMI with pGFP cargo; FP-MEM-pGFP: cells subjected to FP in MEM with pGFP cargo; FP-MtR-pGFP: cells subjected to FP with pGFP cargo in MEM falling into RPMI media; FP-Premix-pGFP: cells subjected to FP with pGFP cargo in RPMI:MEM premixed media. Data are shown for *n* ≥ 2 independent experiments. (∗∗*p* < 0.005, ∗∗∗*p* < 0.001 and ∗∗∗∗*p* < 0.0001). Data are shown as mean ± standard deviation.

Considering the importance of calcium for membrane repair, widely reported in the literature,^19^^,^^23^^,^^24^^,^^30^ we first tested whether the addition of calcium to the transfection media could impact delivery efficiencies. We hypothesized that increased calcium signaling would promote rapid membrane recovery, which may translate into decreased delivery efficiencies in filtroporation experiments. To this end, we performed experiments with Jurkat cells and CD34^+^ bone marrow HSPCs, where additional calcium was introduced in the delivery medium. Addition of 1 mM of CaCl_2_ to the transfection medium caused significant decreases in the delivery efficiency of fluorescent cargo (Figures 3A and 3B), suggesting that membrane repair may be hastened in this setting and that at least one calcium-dependent repair mechanism is involved to reestablish the membrane barrier.

Given that the known membrane repair pathways are calcium dependent, we tested whether calcium signaling was necessary for membrane repair in the context of filtroporation (Figures 3C–3E). We performed FP in calcium-free Minimum Essential Medium (MEM), in addition to FP in our usual delivery medium (Roswell Park Memorial Institute, RPMI), which contains low amounts of calcium (0.42 mM). Since our previous findings indicate that membrane repair takes place within 30 s of poration by filtration, we further investigated whether performing FP in the complete absence of calcium (MEM) could boost delivery efficiency while also mitigating the detrimental effects on viability seen when performing FP in calcium-free phosphate buffered saline (PBS) (Figure 3B). We tested this hypothesis by performing FP in calcium-free MEM and having cells fall into calcium-containing medium (RPMI). Our last experimental condition consisted of a 1:1 mixture of RPMI and MEM to dilute the calcium concentration in RPMI further to test, again, whether this modification could improve delivery efficiency (Figure 3C). We had attempted calcium chelation with several agents (e.g., citrate), but their addition to the culture medium caused massive cell death upon FP (data not shown). Our results showed that untreated controls cultured in MEM with 10% fetal bovine serum (FBS) (MEM10) proliferated at significantly lower rates than those cultured in their usual culture medium (RPMI with 10% FBS, R10) (Figure 3D). Meanwhile, cells filtroporated with MEM and cultured in MEM10 either with or without a green fluorescent protein (GFP)-encoding plasmid (pGFP) cargo (FP-MEM-pGFP or Mock MEM, respectively) did not proliferate over 72 h. Cells in all other conditions were observed to proliferate (cultured in R10) over the 3 days in culture, with the highest rate of proliferation (slope of curve) observed for the Mock RPMI condition (Figure 3D). We found that the highest expression of GFP after delivery of pGFP at low concentration occurred at 24 h post-FP when cells were subjected to FP in RPMI (FP-RPMI-pGFP) with 11 ± 3% of cells expressing GFP at the time of flow cytometry analysis (Figure 3E). We observed no fluorescence when cells were filtroporated in MEM only (FP-MEM-pGFP), as well as no improvement in efficiency when cells were filtroporated in MEM and fell into RPMI (FP-MtR-pGFP, 6 ± 2% GFP^+^), or when MEM and RPMI were premixed (FP-Premix-pGFP, 7 ± 2% GFP^+^). We hypothesized that if calcium is required for membrane repair following FP, cells permeabilized in the absence of calcium (MEM) would not survive for hours following transfection due to their inability to repair the membrane (Mock MEM or FP-MEM-pGFP conditions). However, we do observe cell survival under these conditions, suggesting that cells are either (1) repairing the membrane even in the absence of calcium signaling or (2) that there is a subset of cells that are not permeabilized during FP, likely due to the being of smaller size, such that they can pass through the pores of the filter but are not sufficiently squeezed to require membrane repair. If scenario (2) explains our observations, we would not observe any expression of GFP in the FP-MEM-pGFP population. Indeed, our results show no expression of GFP under these experimental conditions, suggesting that the surviving cells in calcium-free medium were not permeabilized during FP. Furthermore, our findings indicate that performing FP in the complete absence of calcium (FP-MtR-pGFP), or under very low calcium conditions (FP-Premix-pGFP), yielded lower delivery efficiencies than the RPMI conditions. These data indicate that there is a minimum amount of calcium required in the delivery medium at the time of permeabilization that is critical for cell survival, as evidenced by decreased viabilities in the no/lower calcium conditions (FP-RPMI-pGFP: 70 ± 6%; FP-MtR-pGFP: 61 ± 11%; FP-Premix-pGFP: 62 ± 11%) (Figure 3E). This finding suggests that delaying membrane repair has a detrimental impact on cell survival that may mask any positive effects in the delivery of cargoes. If this hypothesis is correct, the observation that MtR conditions yield lower delivery efficiency indicates that membrane repair may be occurring at timescales much shorter than we can investigate in our timed delivery experiments (30 s), given that the timing between filtroporation and contact of cells with the RPMI medium is *ca*. 1 s.

Taken together, these data suggest that calcium signaling is critical for membrane repair following permeabilization by filtroporation and that a subset of cells is not permeabilized during FP. However, we cannot exclude the possibility that cells in the MEM conditions were simply too unhealthy to express GFP, and thus, this could at least partly explain why we do not observe expression. Additionally, spontaneous membrane resealing without calcium signaling may occur when cell membrane injuries are on the order of a few nanometers. Therefore, we also cannot exclude the possibility that pores of that magnitude are present on the cell surfaces in the MEM conditions but are too small to enable plasmid passage.

To elucidate the specific calcium-dependent mechanisms that are involved in resealing the cell membrane after FP, we generated three knockout (KO) cell lines where one protein critical for each of the three aforementioned membrane repair pathways is absent (namely, VAMP7, VPS4B, and GRAF1). We hypothesized that if a particular membrane repair pathway is critical for membrane repair following FP, the absence of a key protein would result in: (a) increased delivery efficiencies, (b) increased mean fluorescent intensity (MFI), or (c) increased cell death in KO cell lines. To test this hypothesis, we performed FP with wild type (WT) Jurkats as controls, along with each of the KO cell lines, delivering FITC-Dex cargo (Figures S6 and S7). We found that GRAF1 KO cells (Figures S6A and S6B), but not VPS4B (Figures S6C and S6D) or VAMP7 KO lines (Figures S6E and S6F), had significantly higher average MFI than WT controls. Interestingly, however, we observed no differences in delivery efficiencies or decreases in cell viability across all KO cell lines when compared to WT Jurkats (Figures S7A–S7C), a finding also seen in nucleofection controls (Figure S7D). To explain this increase in MFI seen only in GRAF1 KO cells, we hypothesize that, because this pathway is reportedly activated within a narrow pore range (50–100 nm), only a small subset of cells had pores within this range after treatments, and thus GRAF1-mediated repair was only required in this subset. Within the population that required GRAF1, membrane repair was impaired or delayed, leading to a greater MFI because the permeabilized cells received greater amounts of fluorescent cargo. However, this subset was likely not a large enough fraction of the total population to result in a statistically significant increase in delivery efficiency overall, although GRAF1 KO cells have slightly higher delivery efficiencies (Figure S7A). Interestingly, and contrary to our hypothesis, we found significant increases in cell viability when VPS4B KO cells were compared to WT Jurkats after FP (Figure S7C), and similarly when GRAF1 KO cells were compared to WT Jurkats in the setting of nucleofection (Figure S7D). Additionally, timed delivery experiments performed with KO cell lines reveal a pattern of membrane repair occurring within 30 s of permeabilization (Figure S8), similar to that seen in WT cells (Figure 2B). These results suggest that membrane repair by inward scission, mediated by GRAF1, is likely involved in repair after FP-mediated permeabilization, but is only active in a subset of cells with pores in the 50–100 nm range. Although no differences were seen in MFI or delivery efficiency for the other two KO cell lines, this observation does not exclude the possibility that the outward budding or patching pathways are also involved in membrane repair following FP as these KO cell lines may be upregulating other pathways and/or other proteins within the same pathway that may be compensating for the loss of the knocked-out protein. This hypothesis is corroborated by the finding that some KO cell lines display higher viabilities, not lower as we initially hypothesized, after treatment by FP and EP.

**Figure 4:**
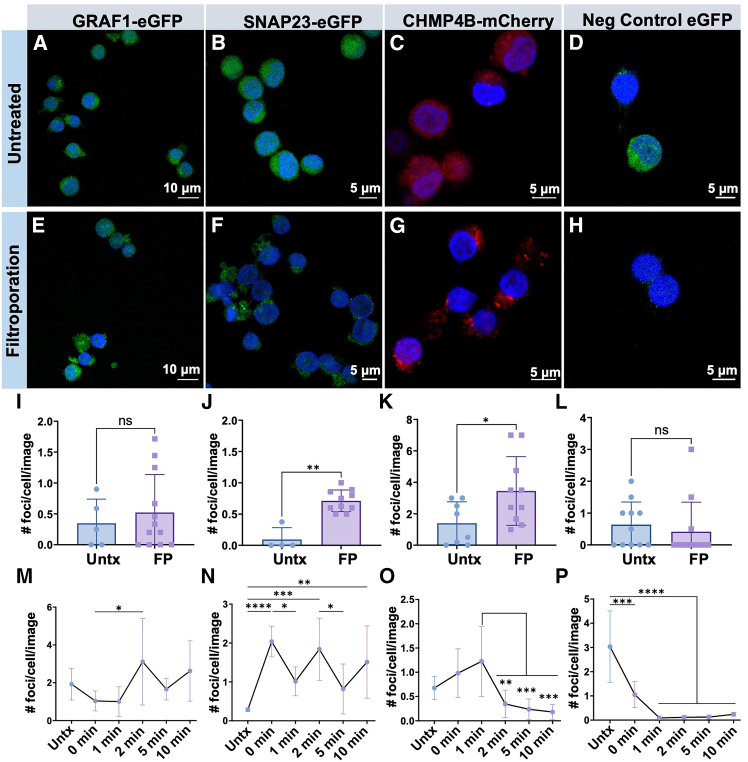
SNAP23 and CHMP4B are involved in membrane repair immediately after filtroporation (A–H) Confocal images overlaying enhanced green fluorescent protein (eGFP) and 4′,6-diamino-2-phenylindole (DAPI) fluorescence channels for untreated and filtroporated Jurkat cells expressing (A,E) GRAF1-eGFP, (B,F) SNAP23-eGFP, and (C,G) CHMP4B-mCherry, with (D,H) free cytoplasmic eGFP as a negative control. (I–P) Quantification of foci per cell per image captured on confocal microscopy for the different cell lines with cells immediately fixed after filtroporation or at different time points (GRAF1-eGFP: I and M; SNAP23-eGFP: J and N; CHMP4B-mCherry: K and O; free cytoplasmic eGFP: L and P). (∗*p* < 0.05, ∗∗*p* < 0.005, ∗∗∗*p* < 0.001, ∗∗∗∗*p* < 0.0001). See also Figures S9–S13. Data are shown as mean ± standard deviation. Scale bars are 10 μm in panels A and E, and 5 μm in all other images.

To visualize membrane repair proteins and to pinpoint their roles in repairing the cell membrane after disruption by FP, we generated reporter cell lines with fluorescent fusion proteins (GRAF1-enhanced GFP (eGFP), SNAP23-eGFP, and CHMP4B-mCherry plus a negative control cell line with free cytoplasmic eGFP) and subjected them to FP and confocal imaging immediately following FP and at various time points post-FP (Figure 4). We observed that filtroporated reporter cell lines fixed immediately displayed increased numbers of foci on the surfaces of the cells (Figures 4I–4L and S9–S12), but only the SNAP23 and CHMP4B cell lines had statistically significant differences (Figures 4B, 4F, 4C, and 4G, respectively). Of note, the negative control displayed very dim fluorescence post-filtroporation due to the leakage of free eGFP from the cytoplasm after permeabilization. In the time course experiments (Figures 4M–4P and S13), we observed that GRAF1-eGFP cells only displayed a statistically significant increase in foci at 2 min post-FP (Figure 4M), suggesting that GRAF1 may participate in membrane repair at a later time, and/or that its involvement is modest because only a subset of cells require GRAF1-mediated repair based on pore size. SNAP23-eGFP displays increased numbers of foci per cell immediately following permeabilization (Figure 4N at 0 min), but, interestingly, foci per cell display a relative drop at 1 and 5 min, and then increase at 2 and 10 min, in a see-saw pattern. Given that SNAP23 is involved in exocytosis, this result may reflect vesicles docking on the surface to repair the membrane in batches. CHMP4B-mCherry cells displayed increases in foci immediately after FP (Figure 4O), which decreased after 2 min. Cytoplasmic eGFP shows a sharp decrease after FP (Figure 4P), as expected, as GFP leaves the cytoplasm when cells are squeezed. These data suggest that all three pathways implicated in membrane repair studied in this work are contributing to cell membrane reconstitution after disruption by FP, although the kinetics and timing of their involvement vary, and that pores in the range of 50 nm up to the micron scale may be created during filtroporation.^24^ These findings also support the results found in other cell-squeezing work.^10^ Of note, RNA-Seq pathway analysis revealed no significantly enriched membrane repair pathways within the FP-treated population (can be searched in Table S3 “FP-enrichment_pathways.xlsx”), suggesting that, at least in HSPCs, membrane repair proteins already present in their cytoplasm are sufficient to repair the defects caused by the permeabilization.

## Discussion

We have developed an economical transfection platform capable of permeabilizing cells by mechanical deformation as cells are pulled through pores of a commercially available track-etched porous membrane by the application of negative pressure. This platform can be assembled from materials commonly available in research laboratories, offering an easily implementable solution for studying intracellular delivery processes.^18^ In the present work, we further elaborated on filtroporation and elucidated critical components of the permeabilization and membrane-repair process. Our RNA-Seq data from HSPCs support filtroporation as a method eliciting fewer transcriptional irregularities, changes in hematopoietic populations, and apoptosis than electroporation, while we show that electroporation may promote lymphoid differentiation and thus might be suitable for cell therapy applications requiring this cell lineage. We show that these changes occur 3 h after treatment, suggesting that early intervention may be required to promote or preclude differentiation. We found that filtroporation promotes cell cycle pathways, even though we have not seen increased cell proliferation following this treatment.^18^ Given that cell cycling is required for homology-directed repair, filtroporation may be a relevant method for gene correction, particularly if donor templates are provided within 3 h of permeabilization. We observed that membrane repair following FP is a rapid process and takes place within seconds of squeezing, as evidenced by significant decreases in transfection efficiency when cargo delivery is delayed. We find similar results in EP-treated cells, where membrane repair also occurs within 1 min of permeabilization by the application of an electric current. The membrane repair literature supports that repair after membrane injury is calcium dependent, and influx of calcium into cells after permeabilization is the key trigger for the restoration of the integrity of the phospholipid bilayer by endogenous proteins.^24^^,^^30^^,^^31^ Here, we find that calcium signaling is critical for membrane repair and survival following FP as well, suggesting that membrane repair occurring after FP is also a calcium-dependent process. Experiments performed with reporter and KO cell lines suggest that three calcium-dependent membrane repair pathways may be involved to reseal discontinuities inflicted by filtroporation, indicating that the pores present range from tens of nanometers to microns. These findings, in turn, inform us about the sizes of cargoes that can be delivered by filtroporation, and thus, the applicability of our platform. The strong dependence on the calcium concentration of the cell culture medium enables us to choose the optimal conditions for filtroporation in order to maximize efficiency and cell survival. This platform can also be applied for generating membrane damage for studying various repair pathways.

### Limitations of the study

One limitation of this current embodiment of filtroporation is the availability of limited pore sizes from commercial vendors, which ultimately limits the cell types that are amenable to permeabilization without massive cell loss. Manufacturing filter membranes with controllable pore size and distribution would enable applications of this platform to any cell type and is thus a promising strategy to universalize filtroporation approaches for cell transfection and membrane repair studies. Information obtained from knockout cell line experiments was also limited, likely due to compensatory membrane repair mechanisms that were not elucidated by the present study. Our confocal microscopy data on reporter fluorescent cell lines were also limited by the resolution of the instruments used, such that multiple protein foci may have been counted as one due to our inability to resolve individual foci.

## Resource availability

### Lead contact

Requests for further information and resources should be directed to and will be fulfilled by the lead contact, Steven J. Jonas (sjonas@ucla.edu).

### Materials availability

Plasmids encoding fluorescently tagged proteins, fluorescent cell lines, and knockout cell lines are available upon request.

### Data and code availability

• RNA Sequencing data have been deposited at the ArrayExpress database (http://www.ebi.ac.uk/arrayexpress) under accession number E-MTAB-15741 and are publicly available as of the date of publication.
• All data reported in this article will be shared by the lead contact upon request.
• Main pathway analysis results are included as supplemental information.
• All original code is available in this article’s supplemental information.
• Files for the 3D printing of filtroporation platforms have been deposited at the NIH 3D repository and are publicly available as of the date of publication at 3DPX-022977.
• Any additional information required to reanalyze the data reported in this article is available from the lead contact upon request.

## Acknowledgments

This research was supported by the 10.13039/100000002National Institutes of Health (NIH) Common Fund through a NIH Director’s Early Independence Award cofunded by the National Institute of Dental and Craniofacial Research and Office of the Director, 10.13039/100000002NIH (Grant DP5OD028181 to S.J.J.). I.M.F. gratefully acknowledges the UCLA Medical Scientist Training Program (Grant T32 GM008042) and the Biotechnology Training in Biomedical Sciences and Engineering (Grant T32 GM067555-11). P.S.W. and S.J.J. thank the Challenge Initiative at UCLA for support. We thank the staff of the BSCRC Flow Cytometry and Microscopy Cores for their help and support. We thank the staff of the UCLA Technology Center for Genomics & Bioinformatics, particularly Prof. Matteo Pellegrini and Dr. Giorgia Del Vecchio, for their assistance with RNA-Seq library preparation, sequencing, and data analysis. We thank Dr. Jae Hyeon Park for assistance with the 3D modeling of vacuum platforms. We thank Prof. Donald Kohn, Dr. Zulema Romero, Dr. Roger Hollis, and Ralph Valentine Crisostomo for assistance and guidance with CRISPR and cloning experiments. We thank Prof. Andrew Goldstein and Matthew Bernard for assistance with Western blot studies. We thank Prof. Norma Andrews for her valuable help and expertise in planning the membrane-repair experiments. Graphical abstract was created using Biorender (Sims, R. (2025) https://BioRender.com/oe0kl4n).

## Author contributions

Experiments were designed by all authors and performed by I.M.F., R.S., A.Y., R.M., M.F., R.A.F., E.D., K.Y.C, and E.S. Microscopy was performed by R.S., R.A.F., and E.D. Raw sequencing data were processed and analyzed by K.J. and the UCLA Technology Center for Genomics and Bioinformatics. RNA Sequencing pathway analysis and data visualization were performed by I.M.F., A.Y., and E.S. The data were interpreted by all authors. The article was written with contributions from all authors. All authors approved the final version of the article.

## Declaration of interests

I.M.F., S.J.J., and P.S.W. have patents and patent applications related to this work. The authors declare no competing interests.

## STAR★Methods

### Key resources table

**Table:** 

REAGENT or RESOURCE	SOURCE	IDENTIFIER
**Antibodies**		

Rabbit polyclonal anti-GRAF1	Novus Biologicals	Cat#NBP1-89732; RRID: AB_11017361
Rabbit polyclonal anti-VPS4B	Bioss	Cat#bs-12778R
Mouse monoclonal anti-VAMP7	Abcam	Cat#ab36195; RRID: AB_2212928
Goat anti-Rabbit IgG	Thermo Fisher Scientific	Cat#A-11010; RRID: AB_2534077
Goat anti-mouse IgG	Thermo Fisher Scientific	Cat#A-31555; RRID: AB_2536171

**Bacterial and virus strains**		

SNAP23-eGFP Lentivirus	This paper	N/A
GRAF1-eGFP Lentivirus	This paper	N/A
CHMP4B-mCherry Lentivirus	This paper	N/A
Cytoplasmic (MNDU)eGFP Lentivirus	This paper	N/A

**Biological samples**		

Human: Cord blood-derived hematopoietic stem and progenitor cells	STEMCELL Technologies	Cat#70008.2, Cat#200-0002, Cat#200-0001
Human: Peripheral blood-derived hematopoietic stem and progenitor cells (G-CSF mobilized)	STEMCELL Technologies	Cat#70060.4, Cat#70060.3
Human: Peripheral blood-derived hematopoietic stem and progenitor cells (Plerixafor and G-CSF mobilized)	STEMCELL Technologies	Cat#70073.3

**Chemicals, peptides, and recombinant proteins**		

StemSpan™ SFEM II	STEMCELL Technologies	Cat#09655
Recombinant Human IL-3	Peprotech	Cat#200-03
Recombinant Human SCF	Peprotech	Cat#300-07
Recombinant Human IL-6	Peprotech	Cat#200-06
Recombinant Human Flt3-Ligand	Peprotech	Cat#300-19
Recombinant Human TPO	Peprotech	Cat#300-18
Trichloro(1H,1H,2H,2H-perfluorooctyl)silane	Sigma Aldrich	Cat#44893
Alt-R® Cas9 Electroporation Enhancer	Integrated DNA Technologies	Cat#1075916
Poly-L-glutamic acid	Sigma-Aldrich	Cat#P4761
Pluronic^TM^ F-68	Thermo Fisher Scientific	Cat#24040032
Fluorescein isothiocyanate–dextran (40 kDa)	Millipore Sigma	Cat#FD40S
Fluoro-Max Dyed Blue Aqueous Fluorescent Particles	Fisher Scientific	Cat#09-980-484

**Critical commercial assays**		

Gibson Assembly Cloning Kit with Competent Cells	New England Biolabs	Cat#E5510S
Platinum SuperFi II Polymerase Master Mix	Thermo Fisher Scientific	Cat#12368050
TransIT®-293 Transfection Reagent	Mirus Bio	Cat#MIR2700
P3 Primary Cell 4D-Nucleofector™ X Kit S	Lonza	Cat#V4XP-3032
SE Cell Line 4D X Kit S	Lonza	Cat#V4XC-1032
RNeasy Mini Kit	Qiagen	Cat#74106
NEBNext Poly(A) mRNA Magnetic Isolation Module	New England Biolabs	Cat#E7490L
NEBNext Ultra II Directional RNA Library Prep with Sample Purification Beads	New England Biolabs	Cat#E7765S
QuickExtract DNA Extraction Solution	Lucigen	Cat#QE09050

**Deposited data**		

3D print files of filtroporation vacuum chambers for multiple parallel experiments (6- and 12-slot)	Frost et al.^18^	NIH3D Repository (3DPX-022977)
RNA-Seq data	This paper	EMBL-EBI (E-MTAB-15741)

**Experimental models: Cell lines**		

Human: Jurkat Clone E6-1	ATCC	TIB-152
Human: Jurkat VAMP7 Knockout	This paper	N/A
Human: Jurkat GRAF1 Knockout	This paper	N/A
Human: Jurkat VPS4B Knockout	This paper	N/A
Human: Jurkat SNAP23-eGFP	This paper	N/A
Human: Jurkat GRAF1-eGFP	This paper	N/A
Human: Jurkat cytoplasmic (MNDU)-eGFP	This paper	N/A
Human: Jurkat CHMP4B-mCherry	This paper	N/A
Human: HEK293T	ATCC	CRL-3467

**Oligonucleotides**		

sgRNA targeting VAMP7: CACACCAAGCATGTTTGGCA	Morrison et al.^33^ (purchased from Synthego)	N/A
sgRNAs targeting GRAF1: AAAGAAAGAATCTCAGCTTC, GAAAGAATCTCAGCTTCAGG	Synthego	N/A
sgRNA targeting VPS4B: TGATAGAGCAGAAAAAACTAA	Szymańska et al.^34^ (purchased from Synthego)	N/A
sgRNA targeting HBB: CUUGCCCCACAGGGCAGUAA	Romero et al.^15^	N/A
Primers for HBB knockout PCR: forward: CCCGTCTTG TTTGTCCCACC	Romero et al.^15^	N/A
Primers for HBB knockout PCR: reverse: AGACACAAG CCCCCTTGAAA	Romero et al.^15^	N/A
Primers for VAMP7 knockout PCR: forward: AGCCTGC TCTTAAGTCTGTTGT	This paper	N/A
Primers for VAMP7 knockout PCR: reverse: CCCCAAC CCTTGACATCGTG	This paper	N/A
Primers for GRAF1 knockout PCR: forward: GGCTGCA GGATTTAATGGGC	This paper	N/A
Primers for GRAF1 knockout PCR: reverse: AAGACACC ATGCAAACCCAGA	This paper	N/A
See Table S1 for additional oligonucleotides.	–	–

**Recombinant DNA**		

Lentiviral backbone plasmid for cloning fluorescent proteins: pCCLc-MNDU3-eGFP	Gifted from Donald Kohn Lab	N/A
pEGFP-SNAP23	Kuster et al.^35^	Addgene, Cat#101914
pLNCX2-mCherry-CHMP4B	Bleck et al.^36^	Addgene, Cat#116923
pMXs-Puro-ARHGAP26 (GRAF1)	Yao et al.^37^	Addgene, Cat#69467
SNAP23-eGFP-MNDU3 plasmid	This paper	N/A
GRAF1-eGFP-MNDU3 plasmid	This paper	N/A
CHMP4B-mCherry-MNDU3 plasmid	This paper	N/A
pmaxGFP	Lonza	From kits: Cat#V4XP-3032 or V4XC-1032

**Software and algorithms**		

Prism 9	GraphPad	https://www.graphpad.com/
FlowJo v10	BD FlowJo	https://www.flowjo.com/flowjo/
Fiji ImageJ v1.51	NIH	https://imagej.net/ij/download.html
ProcessImage.ijm	This paper	N/A
NucleusCounter.ijm	This paper	N/A
FociCounter.ijm	This paper	N/A
FastQC v0.11.9	Simon Andrews	http://www.bioinformatics.babraham.ac.uk/projects/fastqc/
Trim Galore! v0.6.5	Felix Krueger	https://www.bioinformatics.babraham.ac.uk/projects/trim_galore/
STAR package for alignment	Dobin et al.^33^	http://code.google.com/p/rna-star/
Partek Flow	Illumina	https://www.illumina.com/products/by-type/informatics-products/partek-flow.html/
DESeq2	Bioconductor	https://bioconductor.org/packages/release/bioc/html/DESeq2.html
ShinyGO v0.82	Ge et al.^34^	https://bioinformatics.sdstate.edu/go/

**Other**		

Cell culture inserts (8.0 μm, PET)	Falcon Corning	Cat#CLS353182

### Experimental model and study participant details

#### Jurkat clone E6-1

Jurkat cells were purchased from the American Type Culture Collection (ATCC) and used as initially authenticated by the vendor. Clone E6-1 is a derivative of the Jurkat cell line, established from the peripheral blood of a 14-year-old male patient with acute T-cell leukemia. Cell lines generated in this study (knockout and fluorescent reporter cells) were derived from Jurkat E6-1 and cultured under the same conditions as the wild type cells. Cells were cultured in 1× Roswell Park Memorial Institute Medium (RPMI) 1640 with l-glutamine supplemented with 10% fetal bovine serum (FBS) (Gibco) and 1% penicillin-streptomycin (10,000 units/mL penicillin and 10 mg/mL streptomycin) (Gibco), unless otherwise specified. Cells cultured in Minimum Essential Medium (MEM) (Thermo Fisher Scientific, catalog #11380037) were under the same FBS and antibiotic conditions. Cells were maintained in an incubator at 37°C and 5% CO_2_. Cells were periodically tested for mycoplasma contamination and were not further authenticated in our laboratory.

#### CD34^+^ hematopoietic stem and progenitor cell culture

Cord blood-derived and peripheral blood CD34^+^ HSPCs were obtained from human donors purchased from STEMCELL Technologies. For the RNA Sequencing experiments, cells from a single male donor were used. Other experiments involved various batches of cells from multiple donors, likely derived from both males and females. For culturing, cells were thawed and cultured as described by Hoban et al.^32^ Briefly, thawing was performed in Iscove’s Modified Dulbecco’s Medium (IMDM) (Gibco) containing 20% FBS then pre-stimulated for 24 or 48 h in media composed of StemSpan Serum-Free Expansion Medium II (SFEM-II) (STEMCELL Technologies, 09655) supplemented with penicillin/streptomycin/glutamine (P/S/Glu) (diluted 100× for final concentration) (Thermo Fisher Scientific) and recombinant human stem cell factor (rhSCF) (Peprotech, 300-07), human thrombopoietin (Tpo) (Peprotech, 300-18), and recombinant human Flt3-ligand (Flt3-L) (Peprotech, 300-19) to a final concentration of 50 ng/mL. Filtroporation was performed with cells in this medium, and cells were allowed to rest in these conditions. At 24 h post-treatment, cells were transferred to basal bone marrow medium (BBMM). To prepare BBMM, IMDM is supplemented with 20% FBS, P/S/Glu 100× diluted, 0.5% bovine serum albumin (BSA) (Millipore Sigma) and the following cytokines: recombinant human stem cell factor, recombinant human interleukin-3, and interleukin-6 (Peprotech, 200-03 and 200-06, respectively) to a final concentration of 50 ng/mL. Cells were maintained in an incubator at 37°C and 5% CO_2_. Cells were periodically tested for mycoplasma contamination.

#### HEK 293T

HEK 293T cells were purchased from ATCC and used as initially authenticated by the vendor. No information on sex of the originating human subject was available from the vendor. Given that this was a lentiviral producer cell line, we do not expect that lack of knowledge on sex/gender will affect the generalizability of the study. Cells were cultured in 1× Dulbecco’s Minimal Essential Media (DMEM) supplemented with 10% fetal bovine serum (FBS) and 1% penicillin-streptomycin (10,000 units/mL penicillin and 10 mg/mL streptomycin) in an incubator at 37°C and 5% CO_2_. The cell line was not further authenticated in our laboratory. Cells were periodically tested for mycoplasma contamination.

### Method details

#### Device fabrication and operation

Filtroporation devices were prepared as described previously.^18^ Briefly, 15 mL conical tubes (Corning) were pierced with a 20-gauge needle (BD Precision Guide) right below the 1.0 mL line. Perforated tubes were assembled on a 3D-printed filtroporation vacuum chamber with the perforations facing away from the vacuum inlet and the device attached to the biosafety cabinet’s vacuum source with a rubber tubing. Vacuum pressure was measured with a pressure gauge and consistently measured -20” Hg. Cell culture inserts with 8 μm pores (12-well polyethylene terephthalate, PET) (Falcon Corning, 353182) were then placed into the opening of each of the uncapped 15 mL conical tubes within the filtroporation setup.

#### Three-dimensional printing

Vacuum chambers were constructed using a stereolithographic 3D printer (Form 3, Formlabs, F3-PRINTER) with flexible V2 resin (Formlabs, FLFLGR02). Chambers were designed using 3D modeling software, printed, washed in isopropanol (Formlabs Wash chamber, FH-WA-01) for 15 min, dried with nitrogen gas and post-cured (Formlabs Cure chamber, FH-CU-01c) at 60 °C for 15 min. After this post-curing process, supports were removed by hand from the print. The file for printing this model is available upon request.

#### Filter surface functionalization

To functionalize filters with fluorinated silanes, inserts were first air plasma treated at 100 W and 8 standard cubic centimeters per minute (sccm) of air (HPT-200, Henniker Plasma) for 30 s and immediately transferred to a vacuum desiccator with 200 μL of Trichloro(1H,1H,2H,2H-perfluorooctyl)silane (TPFS) (Sigma Aldrich, 44893) placed on a glass slide; the inserts were left under vacuum for 5-6 h. Inserts were then transferred to an oven (20E Lab Oven, Quincy Labs) at 65 °C overnight to promote condensation between the silane and activated PET surfaces.

#### Delivery of fluorescently labeled cargoes by filtroporation

For FITC-Dex experiments, cargo solution was prepared by dissolving powdered 40 kDa FITC-Dex (Millipore Sigma, FD40S) in RPMI to a final concentration of 0.3 mg/mL. The solution was then filtered through a 0.22 μm filter. Knockout and WT cells were grown to a confluency of 800,000 cells/mL with an average diameter of *ca*. 11.5 μm. Cells were pelleted by centrifugation at 90*g* for 10 min, resuspended in RMPI medium or FITC-dextran solution at 10^7^ cells/mL, and applied to cell culture inserts for filtroporation. After application of vacuum, cells were transferred to a 48-well plate and allowed to recover for 20 min at 4 °C. Populations were then diluted in PBS, transferred to 1.5 mL microcentrifuge tubes, and centrifuged at 5000 rpm for 5 min. Filtroporated cells were washed twice with 1× PBS to remove cell media or excess fluorescent dextran, and washed cells were resuspended at approximately 10^7^ cells/mL in PBS for flow cytometry analysis.

For timed delivery experiments, a FITC-Dex solution of 6.0 mg/mL concentration was prepared and sterilized as described above. The filtroporation setup and cell collection/preparation was also as described previously.^18^ Cells to be mixed with FITC-Dex were initially resuspended in 190 μL RPMI and either mixed with 10 μL of 6 mg/mL dextran solution (final concentration 0.3 mg/mL) before filtroporation (incubation control or premixed conditions) or were filtroporated in 190 μL of pure RPMI and 10 μL FITC-Dex was added and mixed into solution at the specified timepoint. Cell recovery and washing in preparation for flow cytometry was performed as described above.

For PS bead experiments, fluorescent beads (Fluoro-Max Dyed Blue Aqueous Fluorescent Particles, Fisher Scientific, 09-980-484) were sonicated to break up aggregates and diluted to 30 μg/mL for filtroporation and nucleofection experiments (stock solution is 1% solids in buffer). Following transfection, cells were washed twice in 1× PBS and plated in 48-well plates for fluorescent microscopy.

#### Delivery of plasmid by filtroporation

For plasmid delivery experiments, cells were resuspended in RPMI and a GFP-encoding plasmid (pmaxGFP, Lonza) was added to a final concentration of 50 μg/mL prior to filtroporation. To test delivery in the absence of calcium (MEM) with cells falling into calcium-containing media (RPMI), 100 μL cell medium was introduced at the bottom of the collection tube while volume of cell suspensions plus cargo were kept at 200 μL. To eliminate any variability attributable to differences in final volumes post-transfection, 100 μL of cell medium was introduced at the bottom of the collection tube for all other conditions as well. Transfection was performed as described above.

#### Delivery of CRISPR/Cas9 reagents by filtroporation

For CRISPR Cas9 ribonucleoprotein (RNP) experiments, RNPs were prepared by mixing the specified amount of Cas9 (Macrolab) (*e.g.*, 300 pmol) with the desired ratio of synthetic modified sgRNA to achieve the desired ratio (*e.g.*, 360 pmol of sgRNA for a 1:1.2 ratio) and incubated on ice for 10 min. Ribonucleoproteins were then added to pre-stimulation medium, as described above, for a total volume of 200 μL. Post-treatment cells were placed in wells of cell culture plates and fresh complete medium was added to reach a final concentration of 500,000 cells/mL. At 24 h, cell viability was measured by AO/PI fluorescent staining and analyzed on a Denovix cell counter (CellDrop FL). Hematopoietic stem and progenitor cells were then centrifuged at low speed (100*g* for 10 min) to remove dead cells and transferred to BBMM at a final concentration of 100,000 cells/mL for the next 48 h prior to counting, genomic DNA extraction, PCR analysis, and Sanger sequencing (Laragen).

When utilized, oligonucleotide enhancer (Alt-R® Cas9 Electroporation Enhancer, Integrated DNA Technologies, 1075916) at the stock concentration of 100 μM was diluted to a 4 μM final concentration for transfection experiments. For experiments containing PGA (Sigma-Aldrich, P4761), the polymer was purchased dry, resuspended to 100 mg/mL in sterile water, and stored at -80 °C prior to use. The PGA solution was thawed prior to use and mixed with sgRNA at the desired volume ratio (*e.g.,* 0.6:1, 0.8:1 or 1:1 v/v) prior to complexing with the Cas9 (Cas9:sgRNA molar ratio of 1:1.2). Pluronic F-68 (Thermo Fisher Scientific, 24040032) was diluted to a final concentration of 1% v/v in cell media.

#### Nucleofection

Fluorescent dextran cargo solution was prepared as described above at a concentration of 6 mg/mL. Two hundred thousand Jurkat cells per condition were pelleted at 90*g* for 15 min and resuspended in nucleofection buffer (SE Cell Line 4D Kit; Lonza, V4XC-1032) following the manufacturer’s instructions (to reach a final reaction volume of 20 μL). For conditions with FITC-Dex, 1 μL of FITC-Dex was added to 19 μL of cells resuspended in SE buffer either prior to nucleofection (premixed condition) or after nucleofection at the specified time point. Cells were transferred to a 16-well strip, allowed to settle for 10 min, and placed in a 4D-Nucleofector (Lonza) with program CL-120 applied. The fluorescent cargo was added directly into the strip for timed delivery experiments. After treatment or after cargo introduction, cells were allowed to rest in the strip wells for 10 min, then 80 μL of 1× PBS was added to the strip well and the entire volume transferred to a microcentrifuge tube. Washing in preparation for flow cytometry was performed as described above.

For experiments with PS beads, particle solutions were prepared as described above and mixed with cells in SE buffer prior to nucleofection.

Ribonucleoproteins were prepared as previously described^18^ at 30 or 100 pmol Cas9 with 36 or 120 pmol HBB-targeting sgRNA, respectively (HBB-targeting sgRNA: CUUGCCCCACAGGGCAGUAA). Nucleofection was performed following published protocols.^32^ Briefly, 200,000 CD34^+^ HSPCs per condition were pelleted at 90*g* for 15 min and resuspended in 20 μL nucleofection buffer (P3 Primary Cell 4D-Nucleofector X Kit by Lonza, V4XP-3032) with or without RNPs. After mixing, HSPCs were transferred to 16-well strip, settled for 10 min and nucleofected in a 4D-Nucleofector (Lonza) using program DZ-100. After treatment, cells rested for 10 min, then 80 μL of pre-stimulation medium was added to the strip well and the entire volume transferred to a cell culture plate containing additional cell medium for a final cell concentration of 400,000 cells/mL. Plates were incubated at 37 °C for the following 72 – 96 h.

#### DNA extraction and sequencing

After at least 48 h post-delivery of RNPs, DNA from at least 100,000 cells was extracted using QuickExtract (QE) DNA Extraction Solution (Lucigen, QE09050). Cells were centrifuged and resuspended in 1 μL of QE for every 10,000 cells. The cell suspension was then placed in a thermocycler (65 °C for 20 min, 95 °C for 10 min, 8 °C for infinity) and DNA was ready for downstream use. For PCR, DNA was diluted 10× in molecular biology grade water and 1 - 20 μL used in protocols. DNA was amplified with primers specific from the region flanking the sgRNA cut site (forward primer 5’-CCCGTCTTGTTTGTCCCACC-3’ and reverse primer 5’-AGACACAAGCCCCCTTGAAA-3’) using Platinum SuperFi II Polymerase Master Mix (Thermo Fisher Scientific, 12368050), run on a 2% agarose gel to check for a single band and submitted for Sanger sequencing (Laragen).

#### RNA sequencing

Total RNA was extracted and purified from HSPCs after cell lysis (∼400,000 cells per condition in triplicates) using a RNeasy Mini Kit (Qiagen, 74106) as described above. Quality of RNA was tested using a tapestation (Agilent 2200 Tapestation, Agilent Technologies), followed by enrichment for mRNA within the total RNA extracts with a NEBNext Poly(A) mRNA Magnetic Isolation Module (New England Biolabs, E7490L). Libraries were prepared for next-generation sequencing (NGS) using the NEBNext Ultra II Directional RNA Library Prep with Sample Purification Beads (New England Biolabs, E7765S) kit following the manufacturer’s protocol which included reverse transcription of mRNA, ligation of adaptors, and PCR enrichment of libraries. After testing samples for quality again using a tapestation, adaptor-ligated samples were pooled and submitted for NGS at UCLA’s Technology Center for Genomics & Bioinformatics (TCGB). Samples were run on a NovaSeq S1 2X50 with a read depth of 35 M reads/sample.

Sequenced samples were then submitted to quality control (QC) with FastQC, reads were trimmed using Trim Galore! package, aligned to the human transcriptome using STAR^33^ and Salmon packages, and then submitted to post-alignment QC. In Partek Flow, read counts were normalized by median ratio for use in DESeq2. All results of differential gene expression analysis utilized the DESeq2 statistical analysis tool and statistical cutoffs of p < 1 x 10^-11^ and fold-change > 2×. Pathway analysis was performed using ShinyGO^34^ v0.82 to identify significant gene ontology terms in biological processes, cellular components, and molecular function.

Enriched pathways with false discovery rate < 0.01 were searched using keywords, counted, and tabulated. For hematopoiesis pathways, the keywords “differentiation,” “lymphoid,” “myeloid,” “T cell,” “lymphocyte,” “B cell,” “erythrocyte,” “basophil,” “eosinophil,” “mast cell,” “NK” and “natural killer cell,” “monocyte,” “plasma cell,” and “macrophage” were used. For cell cycle, the keywords “cell cycle,” “transcription,” “cell division,” “mitosis,” “mitotic,” “chromatid,” and “chromosome segregation” were used. For apoptosis, keywords were “apoptotic,” or “apoptosis,” “cell death,” and “caspase.” For inflammation, keywords were “inflammatory,” “inflammation,” “cytokine,” “chemokine,” and “interleukin.” If a pathway contained multiple keywords within the same category (*e.g.,* “apoptotic cell death”), it was only counted once.

#### Generation of knockout cell lines

Cell lines were generated by CRISPR/Cas9-mediated KO of each gene with the following single guide RNAs (sgRNAs): VAMP7 sgRNA: CACACCAAGCATGTTTGGCA^35^; GRAF1 sgRNAs: AAAGAAAGAATCTCAGCTTC, GAAAGAATCTCAGCTTCAGG; VPS4B sgRNA: TGATAGAGCAGAAAAAACTAA.^36^ All sgRNAs were purchased from Synthego (synthetic modified). Cells transfected with RNPs were cultured for 3 days followed by assessment of KO efficiency in bulk populations by genomic DNA extraction, PCR (sequences can be found in key resources table), Sanger sequencing, and ICE analysis. Cells were subsequently single cell sorted into 96-well round bottom plates with 20% FBS in RPMI with 1% antibiotics and allowed to expand over 2-3 weeks. Once cell pellets were visible at the bottom of a well, cells were resuspended and transferred to progressively larger plates and again subjected to genomic DNA extraction, PCR, sequencing, and ICE analysis to screen for clones with biallelic KOs. One to two selected clones of each cell line were expanded into T-75 cell culture flasks. Finally, cell lysates were collected for western blotting to confirm knocked out proteins were not present.

#### Western blot

To obtain cell lysates, Jurkat cells (wild type controls and knockout populations) were lysed in RIPA buffer (50 mM Tris-HCl pH 8.0, 150 mM NaCl, 1% NP-40, 0.5% sodium deoxycholate, 0.1% SDS, Fisher Scientific, 89901) containing a cOmplete protease inhibitor cocktail tablet (Roche, 4693116001) and Halt Phosphatase Inhibitor (Thermo Fisher Scientific, 78420). Sonication was performed with a sonic dismembrator (Thermo Fisher Scientific, FB120). To perform western blots, protein concentration of lysates was measured using a bicinchoninic acid (BCA) assay (Pierce BCA Protein Assay Kit, Thermo Fisher Scientific, 23227) according to the manufacturer’s instructions. A 4%-12% Bis-Tris gel (Thermo Fisher Scientific, NP0335) was then loaded with master mixes and run at 200 V for 50 min. Proteins were transferred to a polyvinylidene fluoride (PVDF) membrane (Thermo Fisher Scientific, 88520) and total protein was visualized using the SYPRO RUBY protein blot stain (Thermo Fisher Scientific, S11791). Membranes were then submerged in blocking buffer composed of 5% milk (Fisher Scientific, NC9121673) in PBS + 0.1% Tween-20 (PBST) (Thermo Fisher Scientific, J62844.K2) and incubated at room temperature on a slow rocker for 1 h. Blocking buffer was then drained and mixed with primary antibody targeting the protein of interest: rabbit polyclonal anti-GRAF1 (Novus Biologicals, NBP1-89732), rabbit polyclonal anti-VPS4B (Bioss, bs-12778R), or mouse monoclonal anti-VAMP7 (Abcam, ab36195). Membranes were incubated overnight at 4 °C while slowly rocking. The next day, the primary antibody mixture was drained and membranes washed with PBST for 5 min while rocking for 3 cycles. For secondary antibody staining, antibody was added to a fresh solution of 5% milk in PBST at a concentration of 1:1000: goat anti-rabbit IgG (Thermo Fisher Scientific, A-11010) or goat anti-mouse IgG (Thermo Fisher Scientific, A-31555) and incubated at room temperature for 1.5 h while on a slow rocker. Antibody mixture was then drained and discarded, and membranes were washed with PBST for 5 min while rocking for 3 cycles. Presence of proteins was detected via fluorescence.

#### Generation of reporter cell lines

Sequences for fluorescent fusion proteins were purchased from Addgene: SNAP23-eGFP,^37^ CHMP4B-mCherry,^38^ and GRAF1^39^) and cloned into a lentiviral plasmid backbone (pCCLc-MNDU3-eGFP, a gift from Donald Kohn’s lab) by Gibson assembly. Both SNAP23 and CHMP4B were linked to a fluorescent protein in the originating plasmids and thus were simply cloned into the lentiviral backbone after the eGFP sequence within the backbone was excluded by PCR. The GRAF1 protein was cloned into the pCCLc-MNDU3-eGFP backbone with the addition of a linker to create a new GRAF1-eGFP construct. After successful cloning was determined by whole plasmid sequencing, constructs were packaged into lentiviruses by a producer cell line, HEK 293T. Control cytoplasmic eGFP cell lines received backbone plasmid only. To produce lentiviral particles, sterile cloned plasmid preps were combined with transfer plasmid, gag/pol/rre packaging plasmid, a separate rev packaging plasmid, and VSV-G envelope plasmid and transfected into 293T cells by lipofection (TransIT®-293 Transfection Reagent, Mirus Bio, MIR2700). About 20 h after transfection, transfected cells were incubated in DMEM with 10% FBS (D10) containing 10 mM sodium butyrate and 20 mM HEPES for 6–8 h. Cells were then washed with PBS and cultured in fresh D10 for approximately 40 h. Viral supernatants were collected and filtered through a 0.45-μm filter. Viruses were harvested at day 5 and viral soup was used to transduce Jurkat cells. After transduction, fluorescent Jurkats were sorted by flow cytometry.

#### Post-filtroporation imaging

Cells of the SNAP23-eGFP, GRAF1-eGFP, CHMP4B-mCherry, and MNDU-eGFP cell lines were pelleted by centrifugation at 100*g* for 15 min and resuspended in RPMI without FBS at *ca*. 2 million cells per 200 μL of RPMI. Aliquots of 200 μL from each cell solution were added to cell culture inserts on filtroporation devices. Prior to filtroporation, 200 μL of 10% buffered formalin (Fisherbrand™) was added to the conical tubes, ensuring cells fell directly into formalin immediately after filtroporation. The filtroporation devices were otherwise identical to those described above. An untreated condition was prepared by adding 200 μL of cell solution directly to 200 μL of formalin, bypassing filtroporation. For all conditions, cells remained in the formalin for 30 min prior to mounting.

#### Preparation and confocal microscopy

The cell-formalin mixtures were pelleted with a tabletop centrifuge. Supernatant was removed until only a small droplet remained, which was used to resuspend the cells. The cell-formalin droplet was pipetted onto a glass slide (75 mm x 25 mm, Fisherbrand™) sterilized with 70% ethanol and dried with a Kimwipe. One drop of NucRed™ Dead 647 ReadyProbes™ reagent (Thermo Fisher Scientific, R37113) and one drop of ProLong™ Diamond Antifade Mountant (Thermo Fisher Scientific, P36961) were added on top of the cell-formalin droplet. A cover slip (22 mm x 22 mm, Fisherbrand™) was placed on the three droplets and allowed to dry overnight. Confocal microscopy was performed on a Zeiss LSM 880 confocal microscope at 100x resolution and post-processed on ZEN Blue.

Cells from time course experiments were imaged using a 20× water immersion objective on a Nikon AX/AX R NSPARC confocal microscope. Raw images were deconvoluted and denoised using the Nikon software prior to processing in ImageJ.

### Quantification and statistical analysis

Quantification of delivery efficiency by fluorescence was determined by flow cytometry. Flow cytometry data were acquired and processed using a LSR Fortessa cytometer (BD Biosciences). Data were analyzed using FlowJo v10 software (FlowJo, LLC).

Cell viability was measured by AO/PI fluorescent staining and analyzed on a Denovix cell counter (CellDrop FL). Estimation of insertions and deletions (INDELs) from CRISPR RNP experiments was performed using the tracking of INDELs by decomposition (TIDE) analysis tool or Synthego’s inference of CRISPR edits (ICE) tool.

For time course imaging experiments, images were batch-processed and analyzed using Fiji (ImageJ). Fiji Data S1 (“ProcessImage.ijm,” “NucleusCounter.ijm,” and “FociCounter.ijm”) were used to process and analyze the GRAF, CHMP, SNAP, and GFP images. Nuclei and foci counts generated from batch processing were individually checked, and manual adjustments were made and recorded. CHMP4B cell lines were particularly bright so manual counting was performed to avoid overcounting of foci in control images. Images containing large clumps of overlapping cells were excluded from analysis to prevent miscounting of cells.

Statistical analyses were performed using GraphPad Prism 9 software. Population comparisons were performed using a t test (for comparisons between two groups) or one-way ANOVA (for comparisons of three or more groups). Data were presented as mean values +/- standard deviation; all error bars denote standard deviation. Statistical significance was accepted when p < 0.05 unless denoted otherwise. Statistical details of individual experiments can be found in the corresponding figure legends.

## Supplemental information

Document S1. Figures S1–S13 and Table S1

Table S2. Enriched pathways and differentially expressed genes after electroporation from RNA-Seq data, related to Figure 1F

Table S3. Enriched pathways and differentially expressed genes after filtroporation from RNA-Seq data, related to Figure 1F

Data S1. ImageJ macros for counting foci in cell images, related to Figure 4
