# Supplementary material for: Membrane repair following filtroporation-induced cell permeabilization
Source: iScience. 2025 Dec 5;29(1):114317. doi: 10.1016/j.isci.2025.114317 (PMC12774684; doi:10.1016/j.isci.2025.114317)
Supplement: Document S1. Figures S1–S13 and Table S1 [file mmc1.pdf]

iScience, Volume *29*

Supplemental information

Membrane repair following filtroporation-induced cell permeabilization

Isaura M. Frost, Ruby Sims, Anya Yakimenko, Rachel Ma, Maximilian Floridia, Ruth A. Foley, Emily C. Duggan, Kelly Y. Cai, Kelsey Jorgensen, Emily Skuratovsky, Paul S. Weiss, and Steven J. Jonas

REAGENT or RESOURCE	SOURCE	IDENTIFIER
**Oligonucleotides**		
Primers for VPS4B knockout PCR: forward: TACCCCTTCTCATCTGCTGGAC	This paper	N/A
Primers for VPS4B knockout PCR: reverse: ATCCCATCTATTGCACGTGTTG	This paper	N/A
Primer for lentiviral backbone: forward: TAAAGCGGCCAACTCGACG	This paper	N/A
Primer for lentiviral backbone: reverse: GGTGGCGACCGGTGGAT	This paper	N/A
Primer for eGFP portion of the lentiviral backbone (forward): CCACCGGTCGCCACCATGGTGAGCAAGGGCGAGG	This paper	N/A
Primer for inserting SNAP23-eGFP into lentiviral backbone (reverse): GTCGAGTTGGCCGCTTTATTAGCTGTCAATGAGTTTCTTTGCT	This paper	N/A
Primer for inserting CHMP4B-mCherry into lentiviral backbone (reverse): GTCGAGTTGGCCGCTTTATTACATGGATCCAGCCCAGTT	This paper	N/A
Primer for inserting GRAF1 into lentiviral backbone with linker (backbone portion, reverse): GAATTCCGGGGATCTGAGTC	This paper	N/A
Primer for inserting GRAF1 into lentiviral backbone with linker (GRAF1 portion, forward): CTCAGATCCCCGGAATTCATGGGGCTCCCAGCGCT	This paper	N/A
Primer for inserting GRAF1 into lentiviral backbone with linker (GRAF1 portion, reverse): GTCGAGTTGGCCGCTTTAGAGGAACTCCACGTAATTCTCA	This paper	N/A

Table S1. Additional primers used for manufacturing lentiviral plasmid for generation of reporter cell lines, related to key resources table in Methods.

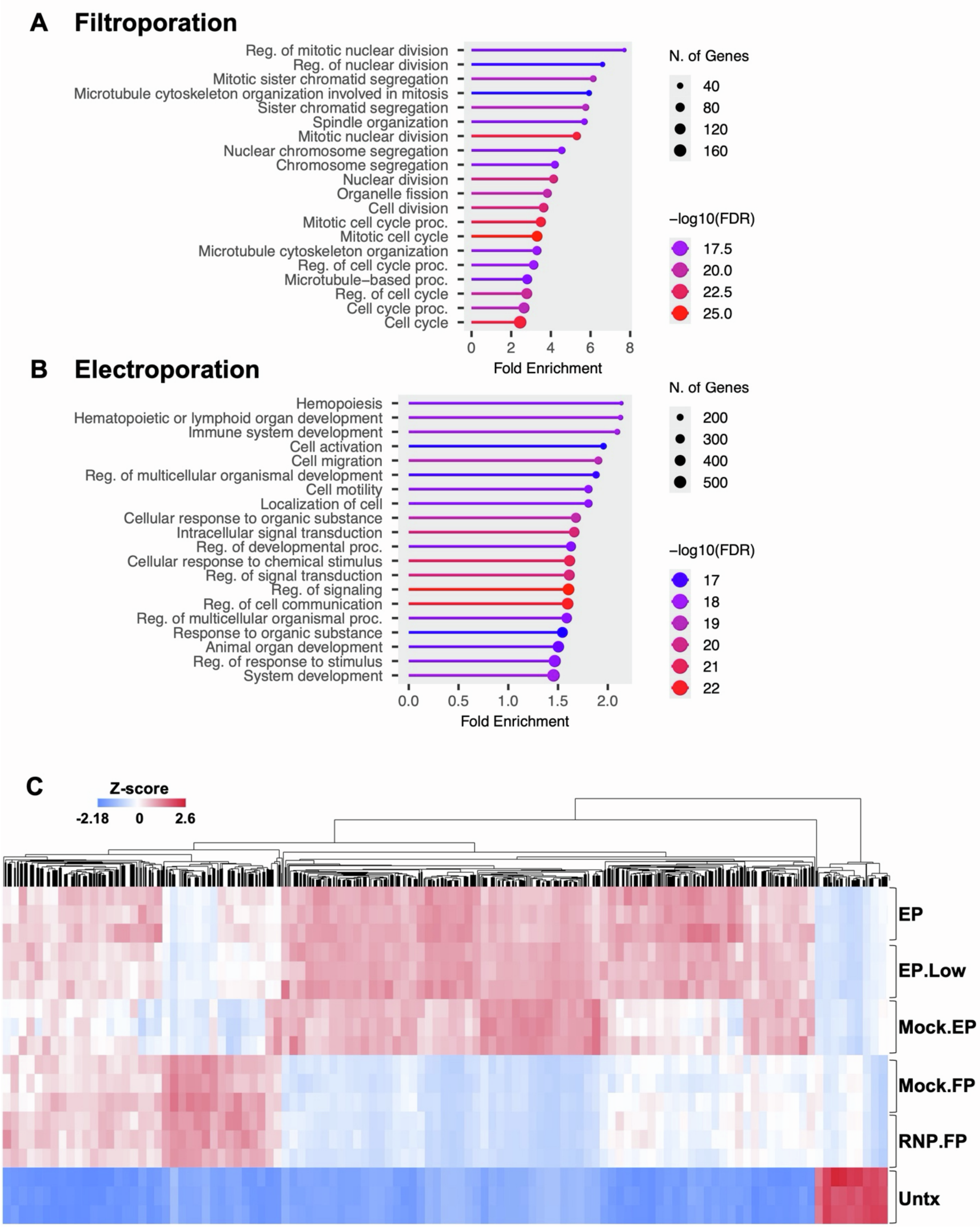
A Filtroporation
Reg. of mitotic nuclear division
Reg. of nuclear division
Mitotic sister chromatid segregation
Microtubule cytoskeleton organization involved in mitosis
Sister chromatid segregation
Spindle organization
Mitotic nuclear division
Nuclear chromosome segregation
Chromosome segregation
Nuclear division
Organelle fission
Cell division
Mitotic cell cycle proc.
Mitotic cell cycle
Microtubule cytoskeleton organization
Reg. of cell cycle proc.
Microtubule−based proc.
Reg. of cell cycle
Cell cycle proc.
Cell cycle
0 2 4 6 8
Fold Enrichment
N. of Genes
40
80
120
160
−log10(FDR)
17.5
20.0
22.5
25.0
B Electroporation
Hemopoiesis
Hematopoietic or lymphoid organ development
Immune system development
Cell activation
Cell migration
Reg. of multicellular organismal development
Cell motility
Localization of cell
Cellular response to organic substance
Intracellular signal transduction
Reg. of developmental proc.
Cellular response to chemical stimulus
Reg. of signal transduction
Reg. of signaling
Reg. of cell communication
Reg. of multicellular organismal proc.
Response to organic substance
Animal organ development
Reg. of response to stimulus
System development
0.0 0.5 1.0 1.5 2.0
Fold Enrichment
N. of Genes
200
300
400
500
−log10(FDR)
17
18
19
20
21
22
C
Z-score
-2.18 0 2.6
EP
EP.Low
Mock.EP
Mock.FP
RNP.FP
Untx

Figure S1. Gene ontology and heat map of differentially expressed genes from RNA Sequencing, related to figure 1. Top 20 enriched biological process pathways from ShinyGO analysis for (A) filtroporation versus untreated and (B) electroporation versus untreated. (C) Heatmap of significantly differentially expressed genes across all conditions from RNA sequencing data. Genes that were significant in both comparisons (EP vs untreated and FP vs untreated) are shown.

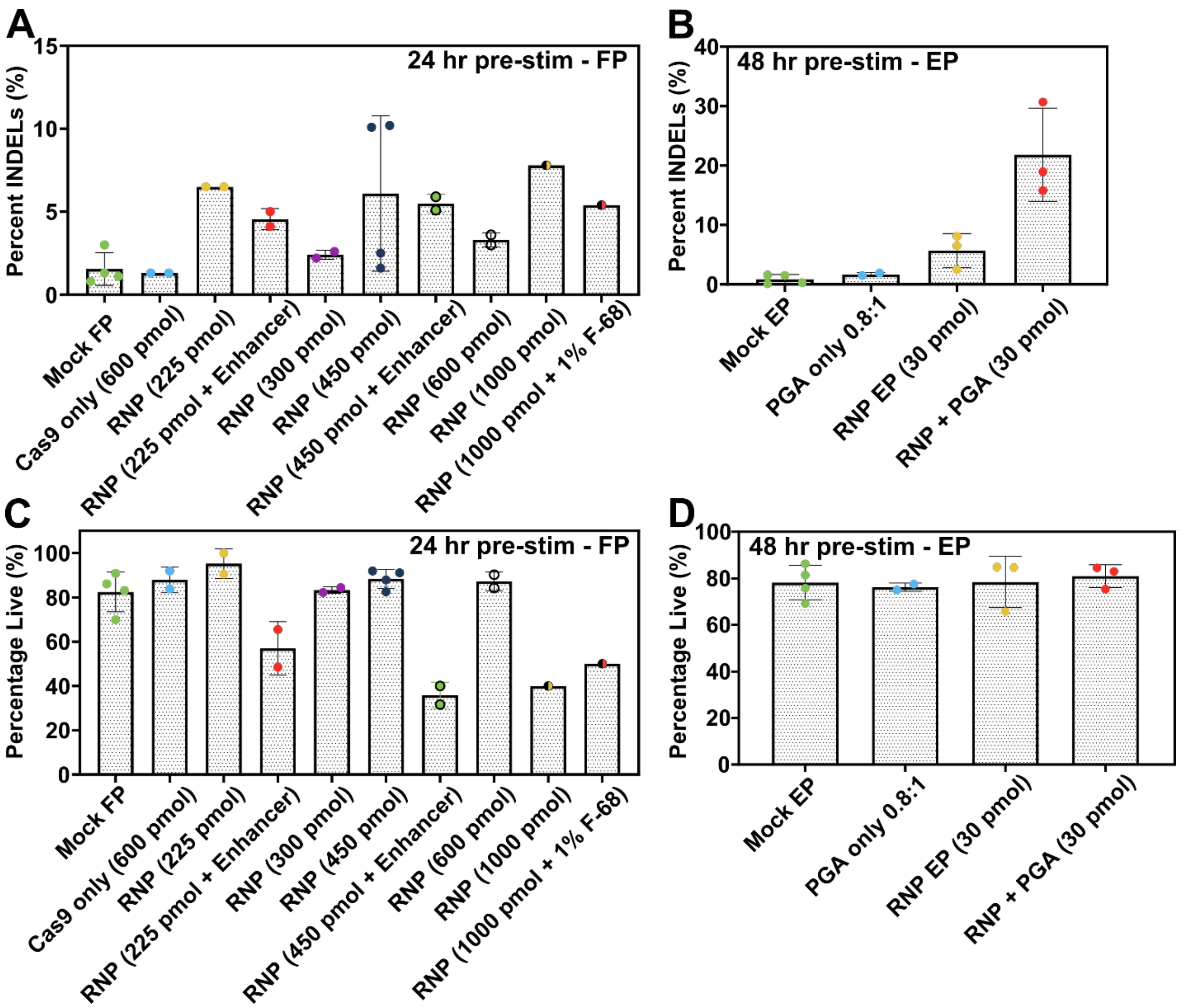

Figure S2. Optimization of ribonucleoprotein (RNP)-mediated editing at the beta globin (HBB) locus in hematopoietic stem and progenitor cells (HSPCs) by filtroporation, related to Figure 1. (A) Percentage of insertions and deletions (INDELs) as determined by PCR, Sanger sequencing, and tracking of INDELs by decomposition (TIDE) analysis of genomic DNA extracted from human hematopoietic stem and progenitor cells (HSPCs) pre-stimulated for 24 h at 4-5 days post-filtroporation (FP) with CRISPR/Cas9 ribonucleoprotein (RNP) with single guide RNA (sgRNA) targeting the beta globin (HBB) gene at various RNP concentrations or in the absence of RNP (Mock FP). (B) Percentage of INDELs in electroporated (EP) HSPCs. (C) Viabilities of HSPC populations 24 h post-FP for conditions in (A) as determined by acridine orange/propidium iodide staining. (D) Viabilities of HSPC populations 24 h post-EP for conditions in (B) as determined by acridine orange/propidium iodide staining. Mock EP: electroporation without cargo; Cas9 only: FP with Cas9 only, no sgRNA, at the indicated concentration; RNP + Enhancer: FP with RNP plus electroporation enhancer; RNP + 1% F-68: FP with RNP plus Pluronic F-68 at 1% v/v concentration; PGA only 0.8:1: EP with PGA only without RNP at 0.8:1 PGA:sgRNA volume ratio; RNP EP: EP with indicated amount of RNP. Data represented as mean values ± standard deviation.

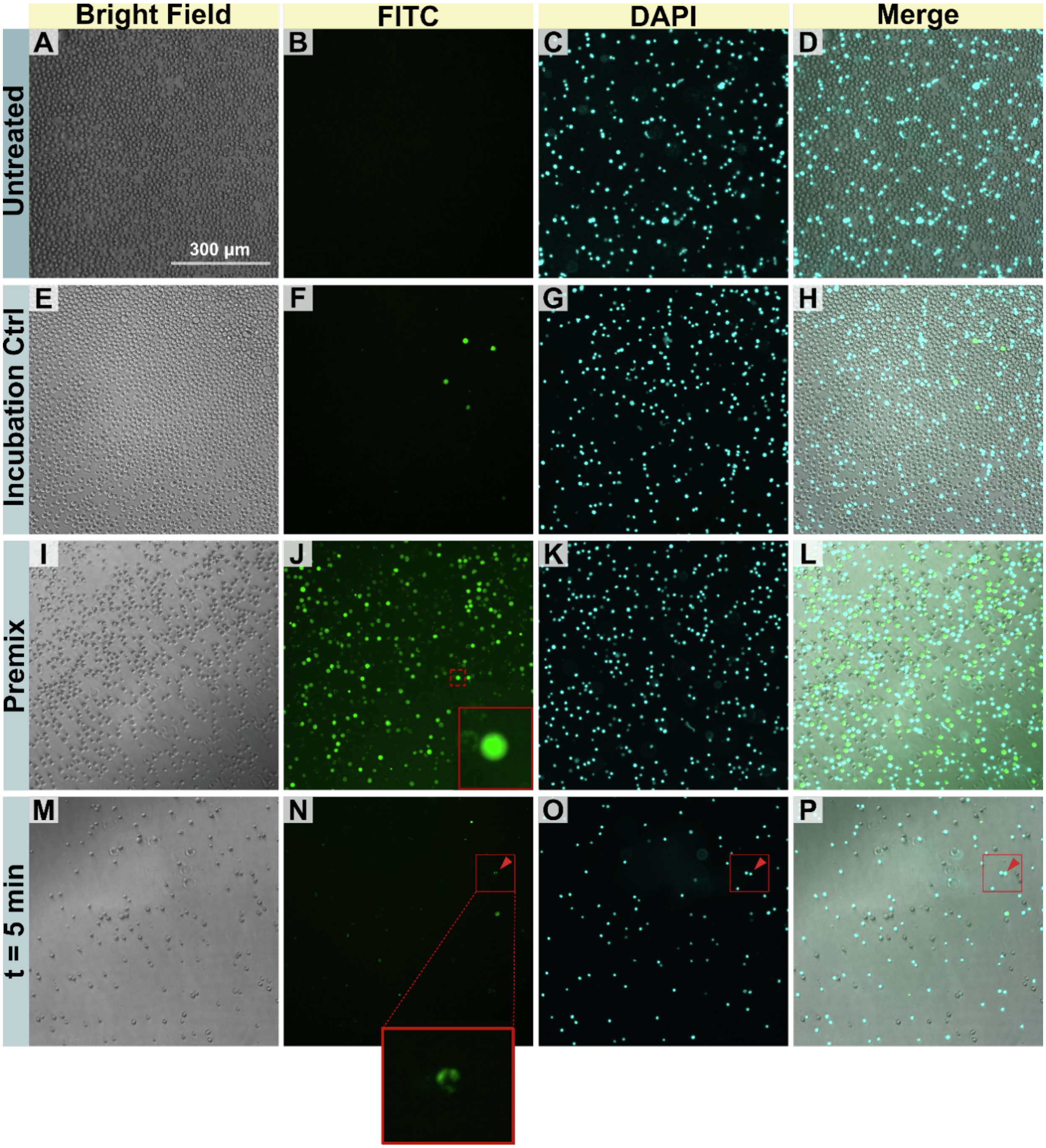

Figure S3. Fluorescent microscopy of Jurkat cells subjected to timed delivery experiments, related to Figure 2. (A-D) Fluorescent microscopy of untreated Jurkat cells live stained with 4',6-diamidino-2-phenylindole (DAPI) for viability. (E-H) Incubation controls were mixed with fluorescent cargo but not filtroporated. (I-L) Cells were premixed with fluorescent cargo fluorescein isothiocyanate (FITC)-tagged dextran (FITC-Dex) and subjected to filtroporation. Inset shows uniformly fluorescent cells in premixed conditions. (M-P) In timed delivery experiments, cells were subjected to filtroporation and cargo was added at 5 min. Areas marked where FITC signal overlapped with DAPI staining indicates dead cells (red arrow). Scale bar is 300 μm.

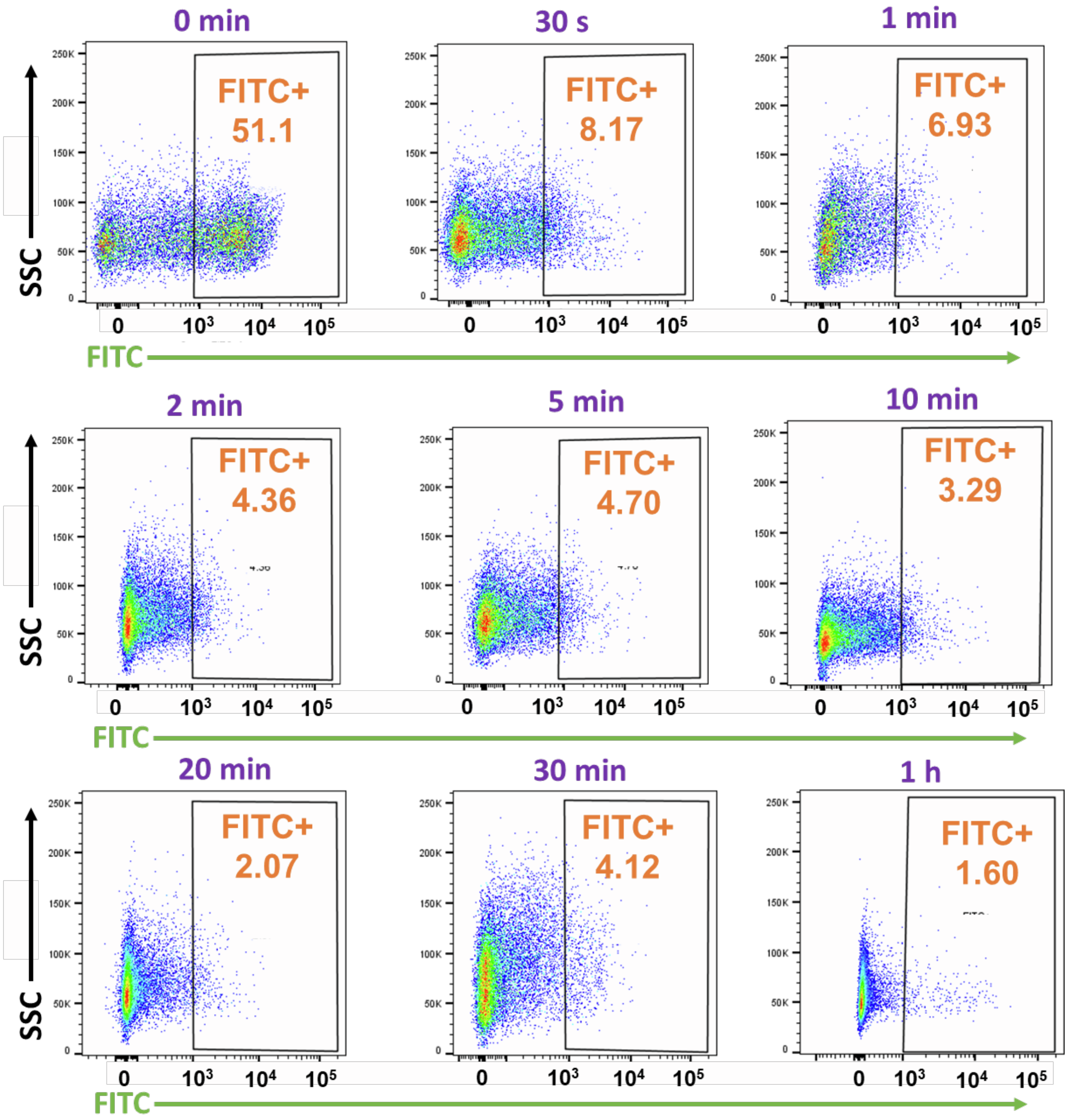

Figure S4. Representative gating strategies for timed delivery experiments, related to Figure 2. Wild-type Jurkat cells were subjected to filtroporation either in the presence of fluorescein isothiocyanate (FITC)-tagged Dextran (0 min) or filtroporated without cargo with cargo introduced at the specified timepoint. The percentage of cells displaying fluorescence was measured by flow cytometry after exclusion of dead cells by 4',6-diamidino-2-phenylindole (DAPI) staining.

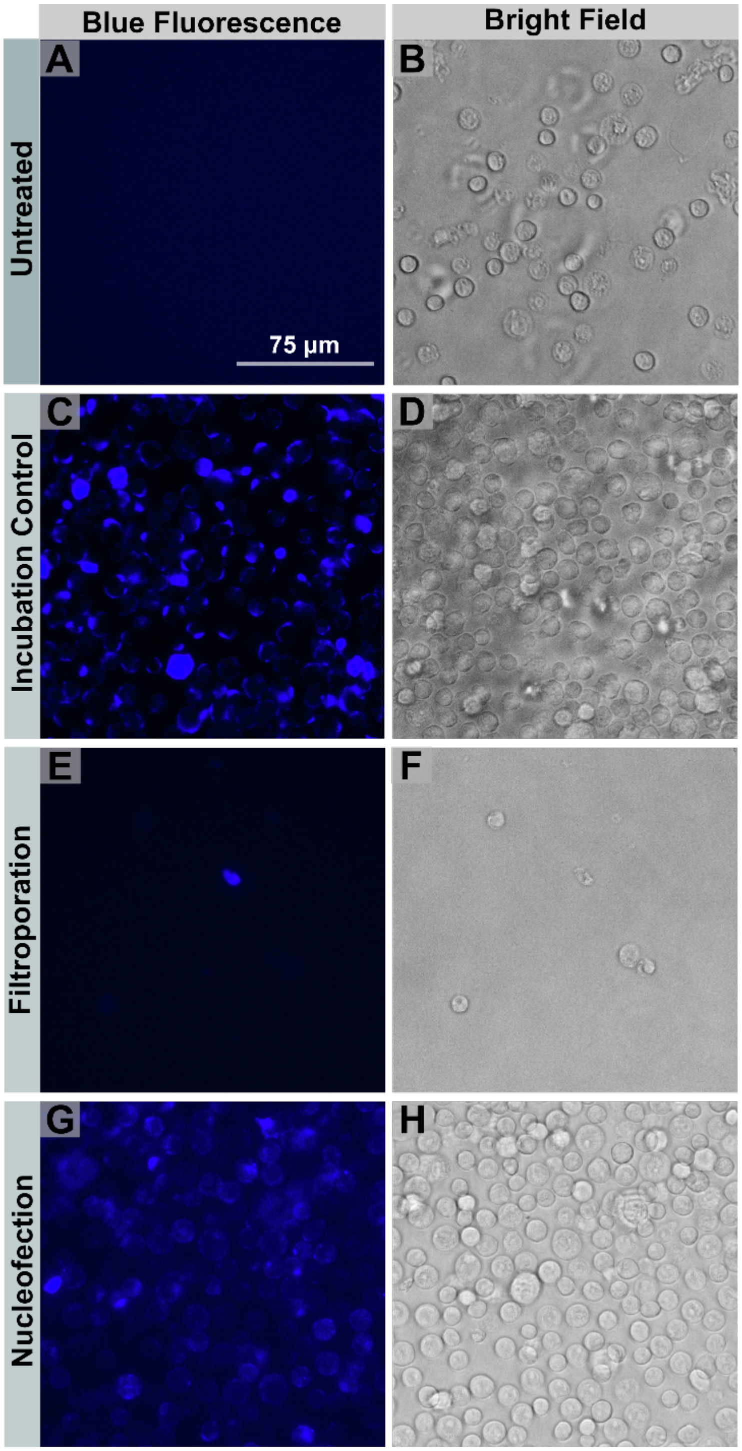

Figure S5. Filtroporation and nucleofection of fluorescent beads in Jurkat cells to probe transfection-induced pore size, related to Figure 2. (A,B) Untreated controls. (C,D) Incubation controls were incubated with fluorescent beads but not subjected to filtroporation. (E-H) Jurkat cells were either (E,F) filtroporated or (G,H) nucleofected with fluorescent polystyrene beads of 50 nm. Images were acquired within 2 h of treatment after washing cells. Scale bar is 75 μm.

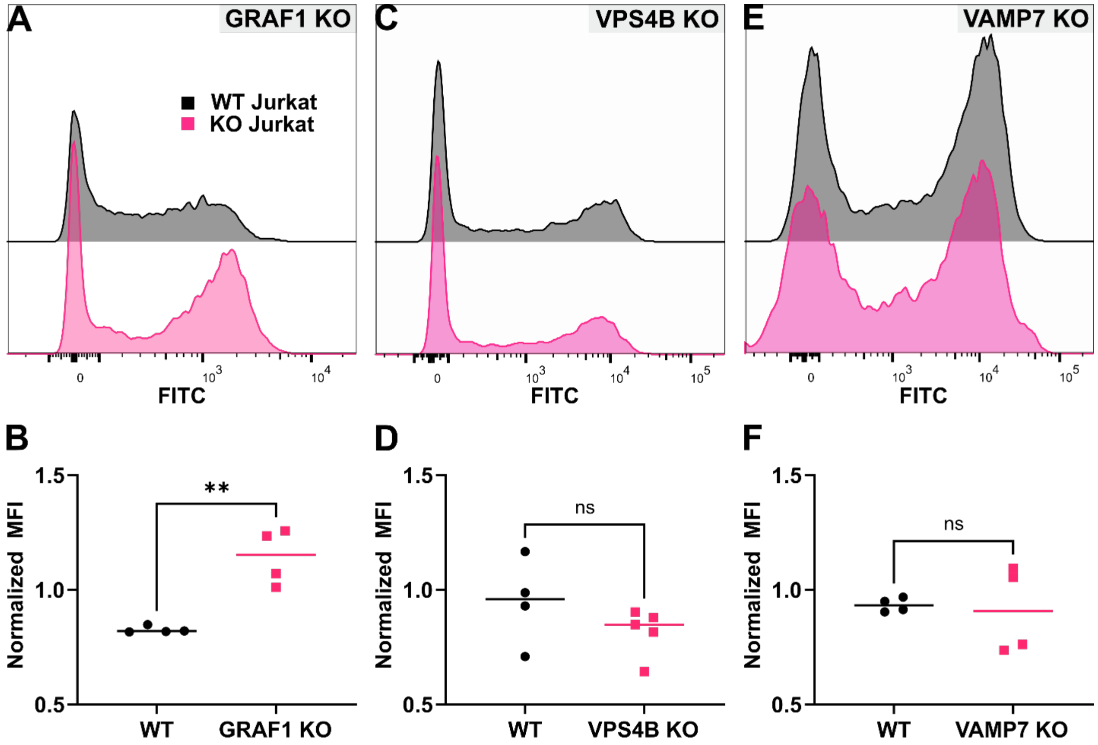

Figure S6. Mean fluorescent intensity (MFI) in filtroporated Jurkat knockout (KO) and wild type (WT) cells, related to Figure 2. (A, C, E) Representative histogram plots of (A) WT and GRAF1 KO, (C) VPS4B KO, or (E) VAMP7 KO cells filtroporated with fluorescein isothiocyanate (FITC)-dextran and analyzed by flow cytometry. (B, D, F) Mean fluorescent intensity (MFI) of FITC$^+$ populations normalized by respective group average. GRAF1: GTPase regulator associated with focal adhesion kinase 1; VAMP7: vesicle associate membrane protein 7; VPS4B: vacuolar protein sorting-associated protein 4B. Data are shown for N = 4 independent experiments. (**P < 0.005).

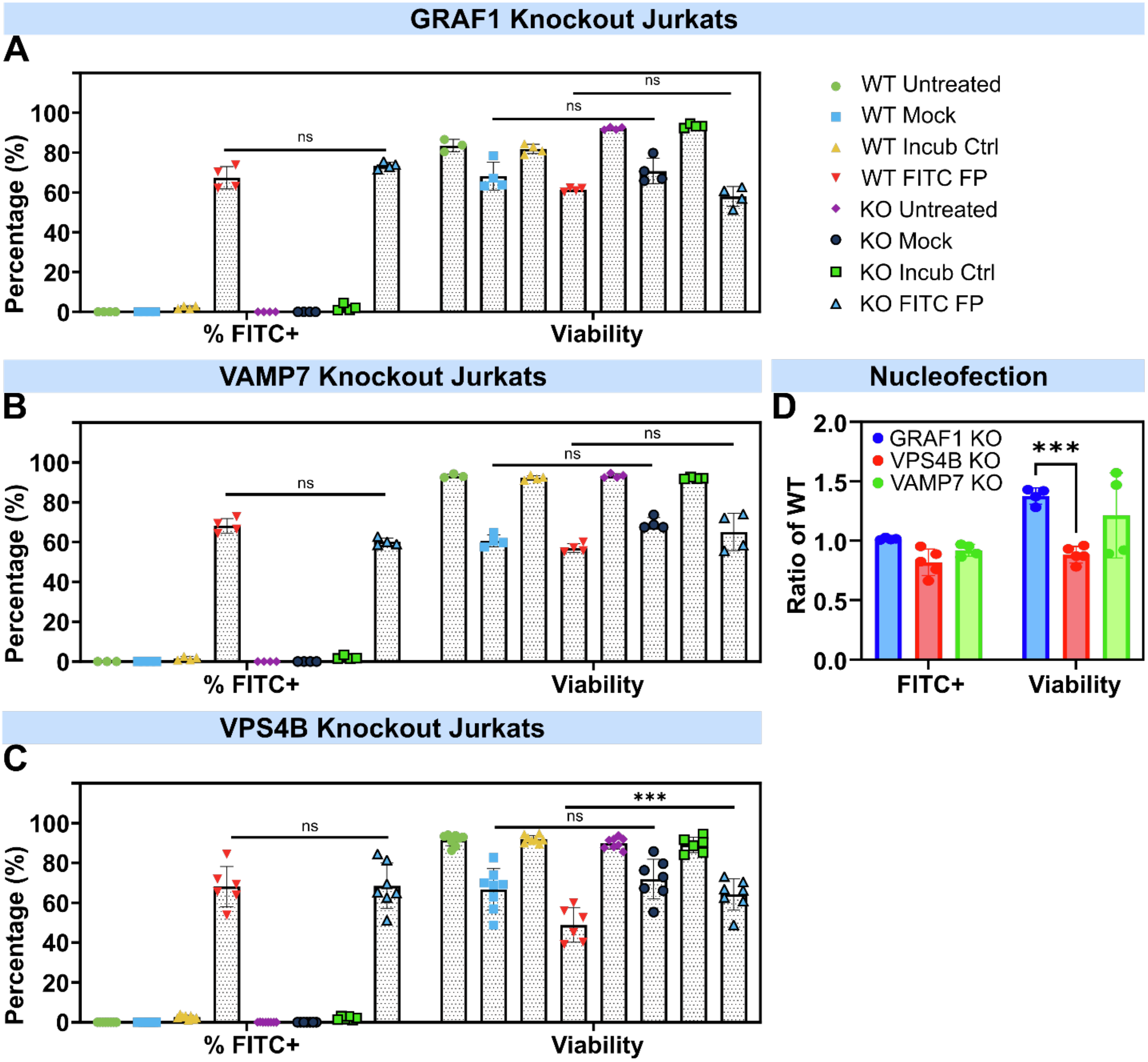

Figure S7. Comparison of filtroporation delivery efficiencies of wild type (WT) Jurkats versus knockout (KO) cell lines, related to Figure 2. (A) Wild type and GRAF1 KO cell lines were subjected to filtroporation (FP) in the presence of fluorescein isothiocyanate (FITC)-tagged dextran; percentage FITC$^+$ cells were measured by flow cytometry (left) while viabilities were measured by 4',6-diamidino-2-phenylindole (DAPI) staining at the time of flow cytometry (right). (B) Wild type and VAMP7 KO cells and (C) WT and VPS4B KO cells were subjected to FP as in (A). The following controls were performed for WT and KO cells: untreated, mock filtroporated (FP without cargo), incubated in FITC dextran without FP (Incub Ctrl). (D) Nucleofection of WT and KO cell lines was also performed as a control; efficiency and viability data was normalized to results obtained with WT cells performed simultaneously. All flow cytometry experiments were performed within 2 h of treatments after cells were washed to remove excess fluorescent cargo. GRAF1: GTPase regulator associated with focal adhesion kinase 1; VAMP7: vesicle associate membrane protein 7; VPS4B: vacuolar protein sorting-associated protein 4B. Data are shown for N ≥ 2 independent experiments. (***$P < 0.001$). Data represented as mean values ± standard deviation.

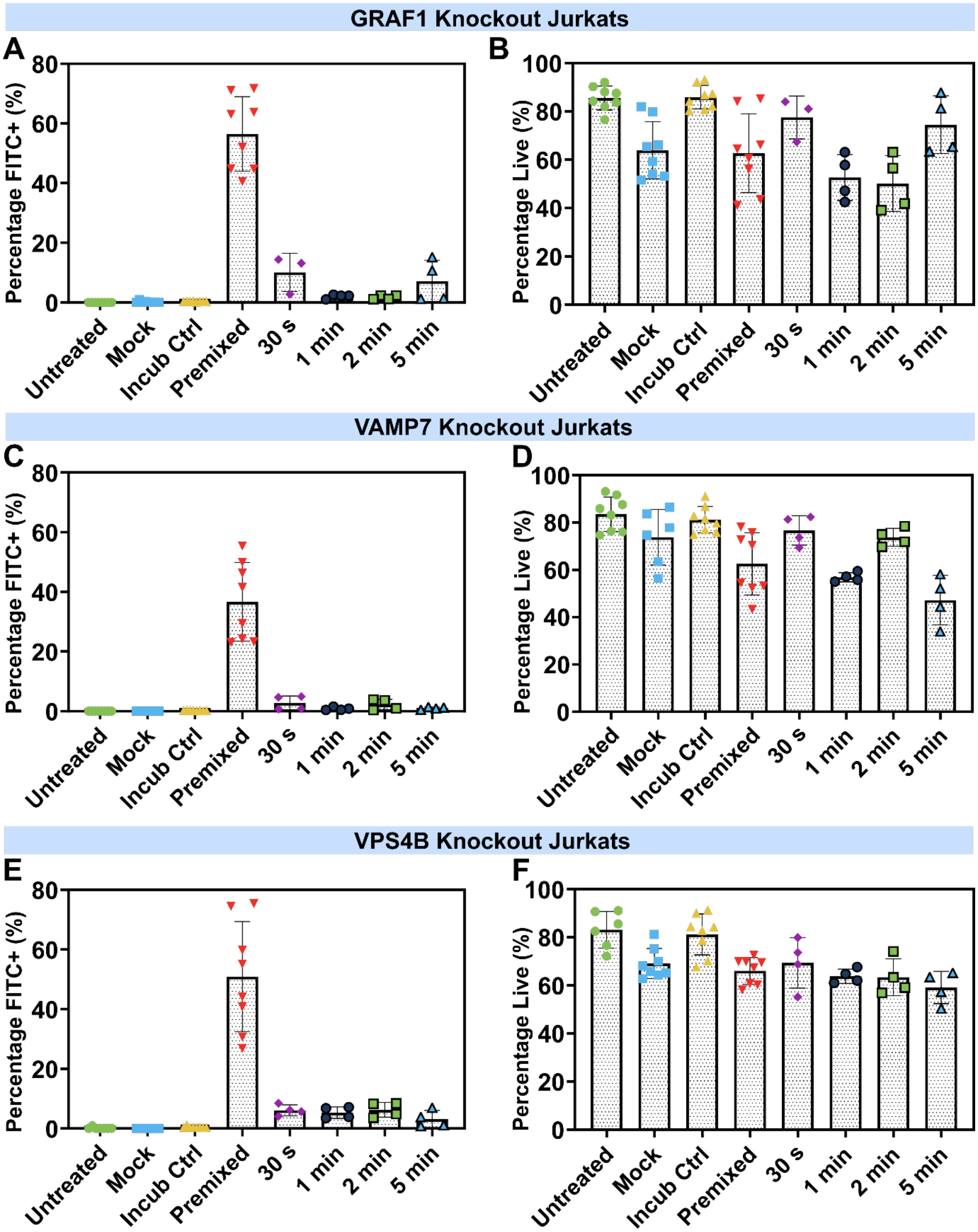

Figure S8. Timed delivery of fluorescent cargo to knockout (KO) cell lines, related to Figure 2. (A, C, E) KO cells were filtroporated (FP) either premixed with fluorescein isothiocyanate (FITC)-tagged dextran or had fluorescent cargo added at the specified time point. (B, D, F) Viabilities determined by 4',6-diamidino-2-phenylindole (DAPI) staining at the time of flow cytometry after FP experiments. All flow cytometry experiments were performed within 2 h of treatments after cells were washed to remove excess fluorescent cargo. Controls were untreated, filtroporated without cargo (Mock) or incubated with FITC-Dex without FP

(Incub Ctrl). GRAF1: GTPase regulator associated with focal adhesion kinase 1; VAMP7: vesicle associate membrane protein 7; VPS4B: vacuolar protein sorting-associated protein 4B. Data are shown for $N \geq 2$ independent experiments. Data represented as mean values ± standard deviation.

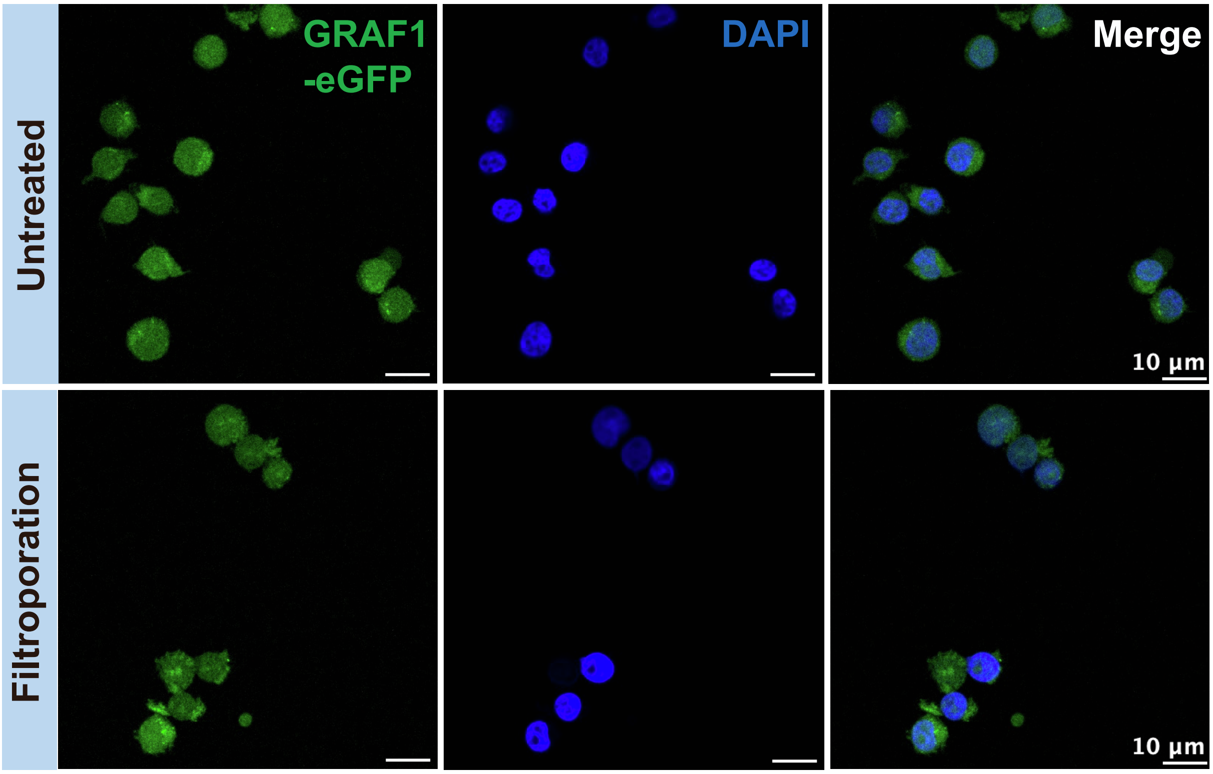

Figure S9. **Individual channels for GRAF1-eGFP cell line micrographs, related to Figure 4.** GRAF1-eGFP (enhanced green fluorescent protein) reporter Jurkat cell line imaged (top row) without treatment or (bottom row) after filtroporation. Confocal imaging was performed using (left column) green channel for GRAF1-eGFP fusion protein imaging, or (middle column) blue channel for 4',6-diamidino-2-phenylindole (DAPI) nuclear imaging. Scale bar is 10 μm.

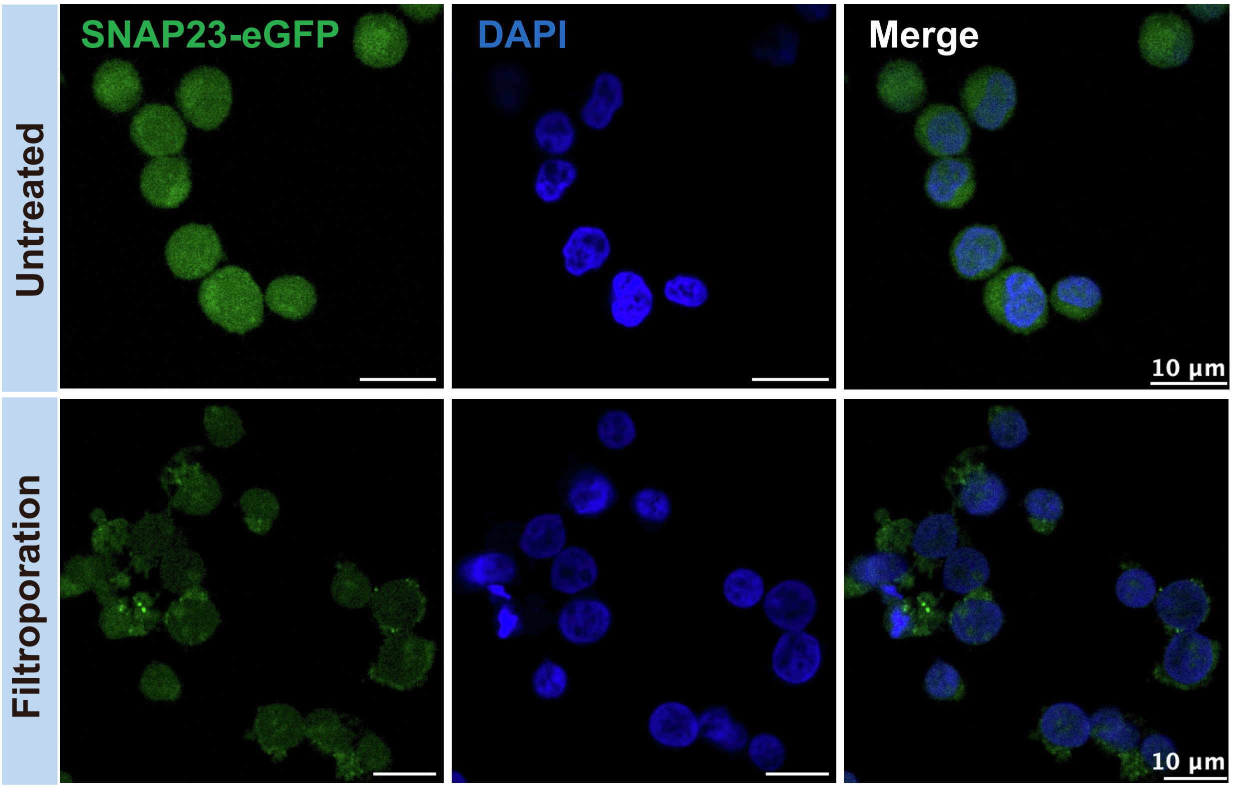

Figure S10. **Individual channels for SNAP23-eGFP cell line micrographs, related to Figure 4.** SNAP23-eGFP (enhanced green fluorescent protein) reporter Jurkat cell line imaged (top row) without treatment or (bottom row) after filtroporation. Confocal imaging was performed using (left column) green channel for SNAP23-eGFP fusion protein imaging, or (middle column) blue channel for 4',6-diamidino-2-phenylindole (DAPI) nuclear imaging. Scale bar is 10 μm.

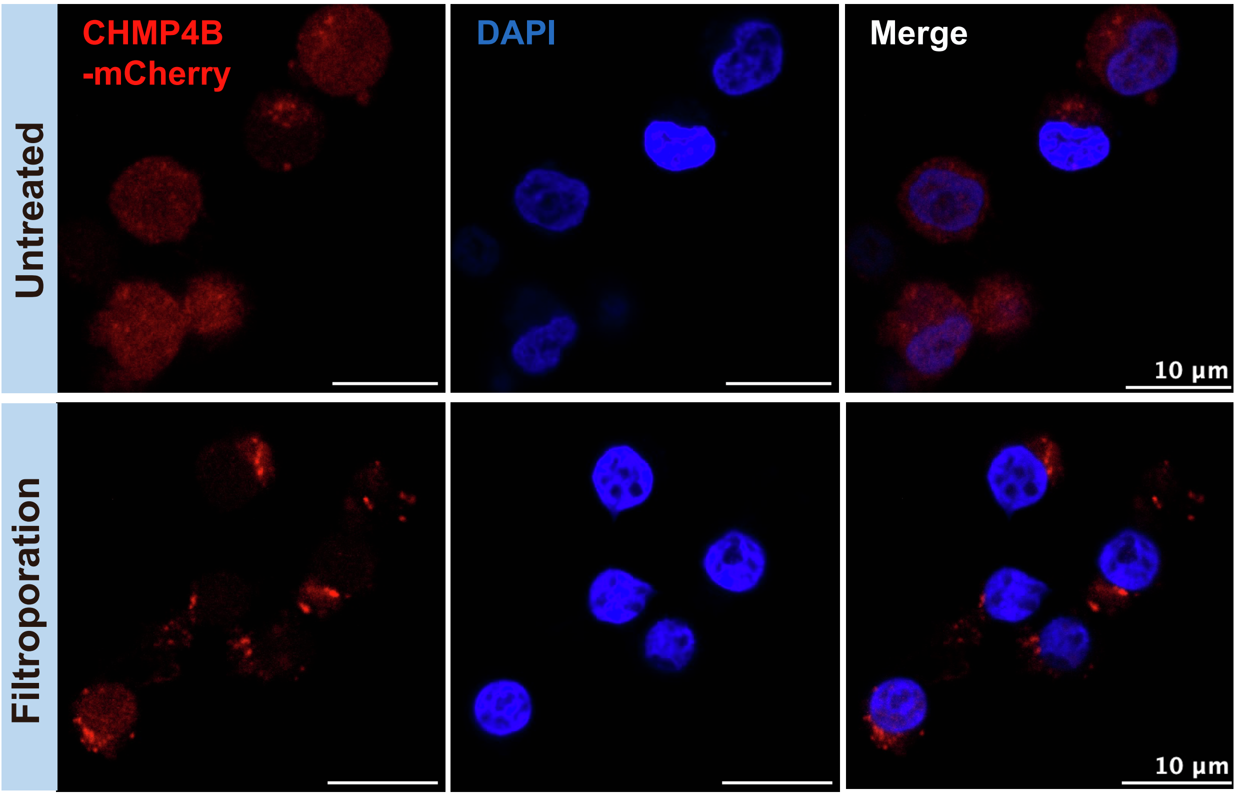

Figure S11. Individual channels for CHMP4B-mCherry cell line micrographs, related to Figure 4. CHMP4B-mCherry reporter Jurkat cell line imaged (top row) without treatment or (bottom row) after filtroporation. Confocal imaging was performed using (left column) red channel for CHMP4B-mCherry fusion protein imaging, or (middle column) blue channel for 4',6-diamidino-2-phenylindole (DAPI) nuclear imaging. Scale bar is 10 μm.

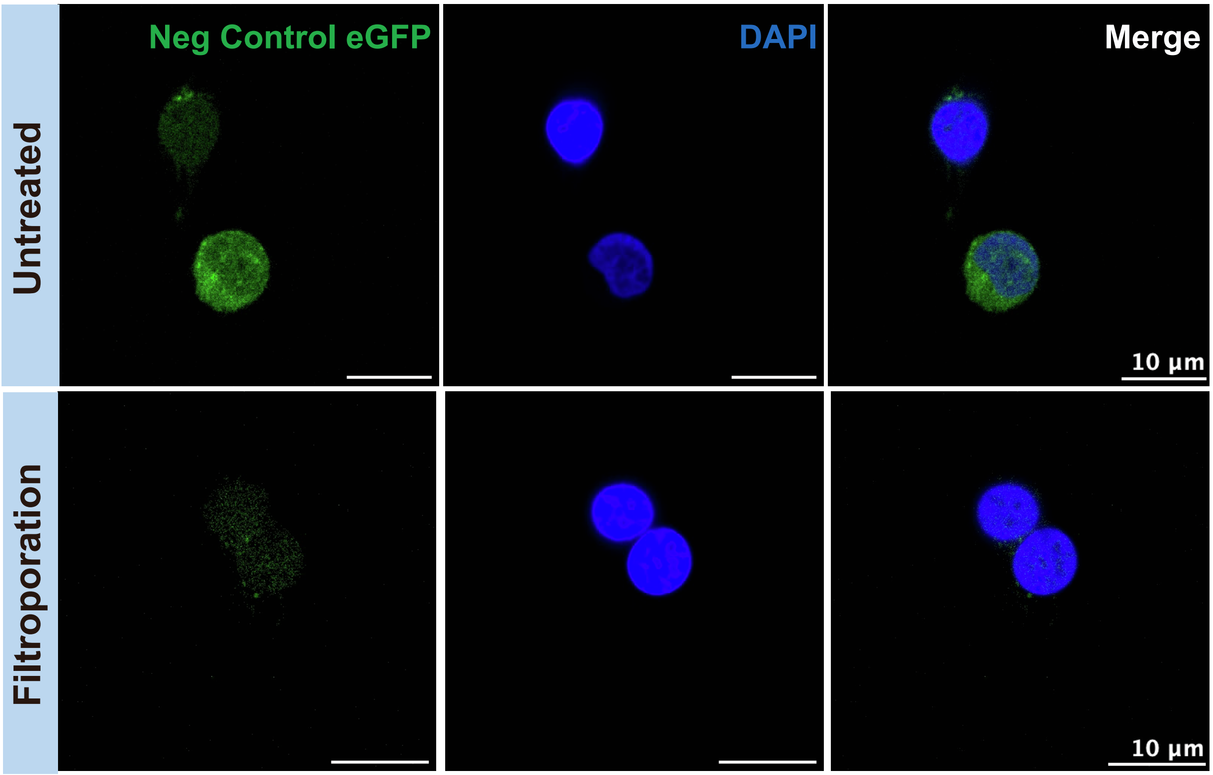

Figure S12. **Individual channels for cytoplasmic eGFP cell line micrographs, related to Figure 4.** Free cytoplasmic enhanced green fluorescent protein (eGFP)-expressing Jurkat cell line imaged (top row) without treatment or (bottom row) after filtroporation. Confocal imaging was performed using the (left column) green channel for eGFP imaging or the (middle column) blue channel for 4’,6-diamidino-2-phenylindole (DAPI) nuclear imaging. Scale bar is 10 µm.

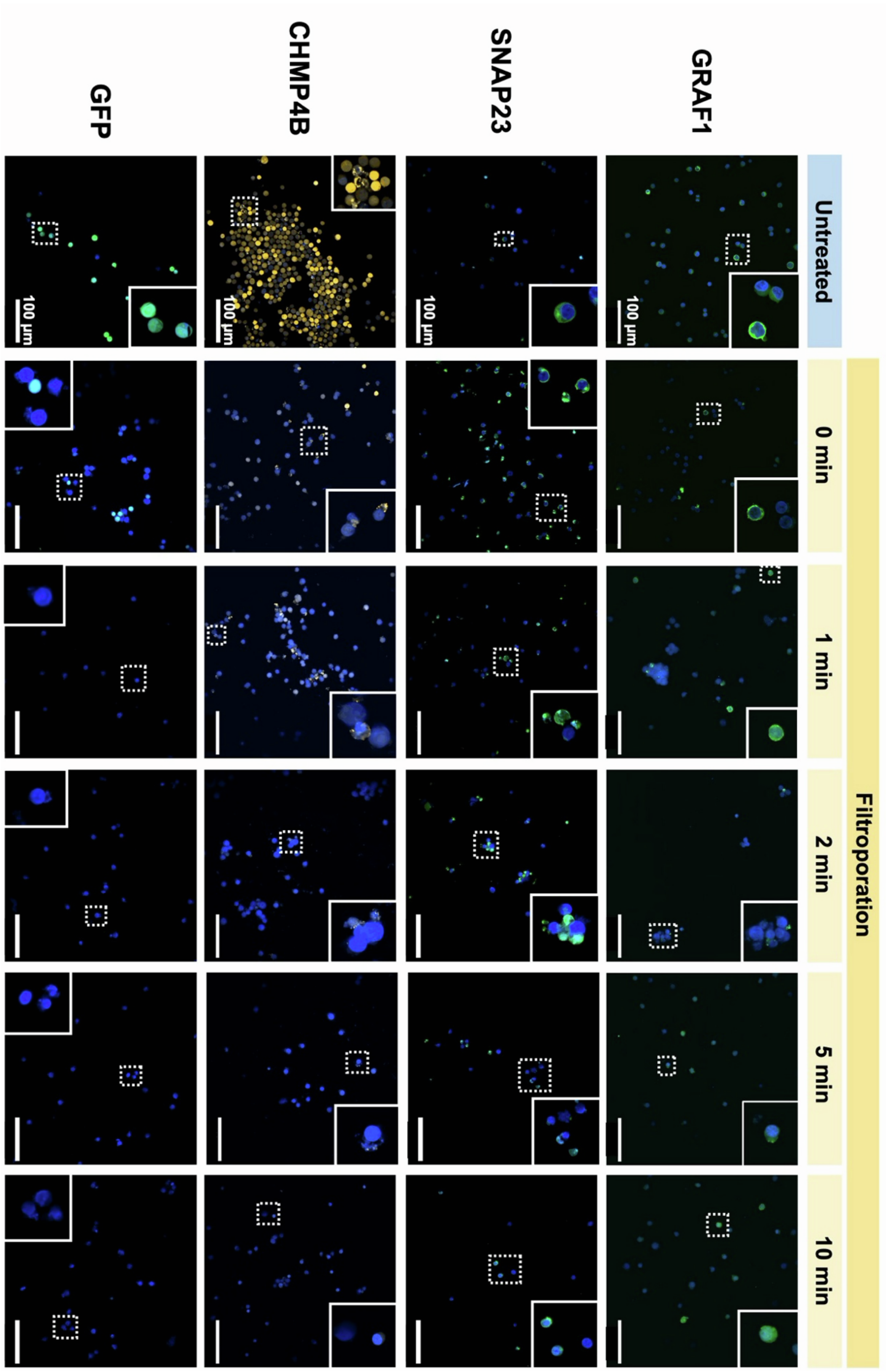

Figure S13. Time course experiment of fluorescent cell lines GRAF1-eGFP, SNAP23-eGFP, CHMP4B-mCherry, and cytoplasmic GFP control, related to Figure 4. Cells were subjected to

filtroporation and fixed at the specified time points, then imaged by confocal microscopy. For GRAF1, SNAP23, and cytoplasmic GFP, confocal imaging was performed using the green channel, while the red channel was used for CHMP4B images. The blue channel was used for 4',6-diamidino-2-phenylindole (DAPI) nuclear imaging. Scale bar is 100 μm.
